# Solid lipid nanoparticles enhance piracetam’s neuroprotective action in streptozotocin-induced cognitive dysfunction

**DOI:** 10.1186/s11671-026-04528-3

**Published:** 2026-03-29

**Authors:** Abhishek Mishra, Bhabani Sankar Satapathy, Pratap Kumar Sahu

**Affiliations:** 1 School of Pharmaceutical Sciences, Siksha ‘O’ Anusandhan (Deemed to Be University), Bhubaneswar, Odisha India; 2 GITAM School of Pharmacy, GITAM Deemed to Be University, Hyderabad,, Telangana India

**Keywords:** Piracetam, Solid-lipid nanoparticle, Diabetes-induced cognitive dysfunction, *In vivo* efficacy

## Abstract

Diabetes-induced cognitive dysfunction remains an unaddressed medical concern across the globe with limited options left with conventional drug therapy. Piracetam, the first identified nootropic agent has been repurposed in the current study via solid-lipid nanoparticle against streptozotocin-induced cognitive dysfunction in rats. Experimental piracetam loaded solid-lipid nanoparticles (PSLNs) were developed by conventional nanoprecipitation method and characterized in terms of SEM, AFM, DLS, zeta potential, drug loading efficiency etc. *In vivo* efficacy of the selected PSLNs was analysed. Various biochemical parameters were analysed along with histopathology and brain pharmacokinetic studies. Experimental PSLNs were spherical, nanosized, homogenous (PDI: 0.21 ± 0.5) with − 38.2 ± 0.3 mV surface charge. Following administration, selected PSLNs brought significant improvement in behavioural and memory function as compared to plain piracetam and control rat groups. Modulation in key enzyme/ protein expression in terms of SOD, CAT, GSH was observed *in vivo* in PSLNs treated rats. Histopathology of brain tissue in AD rats indicated revival of normal tissue architecture in PSLNs treated group as compared to piracetam/control groups. Key brain pharmacokinetic parameters like AUC, AUMC, MRT etc. were substantially improved for PSLNs than plain piracetam, signifying higher accumulation and retention of the drug following nanoencapsulation. However, futuristic studies are necessary to establish PSLNs as a potent modality against diabetes-induced cognitive dysfunction in clinical settings.

**Supplementary Information:**

The online version contains supplementary material available at 10.1186/s11671-026-04528-3.

## Introduction

Diabetes-induced cognitive dysfunction is frequent but has been largely underrated and unaddressed medical condition till today [1]. Out of numerous consequences of diabetes mellitus, cognitive dysfunction and progression of Alzheimer’s disease (AD) has been identified as major neurodegenerative disorder with limited therapeutic options [2, 3]. AD is distinguished by progressive neurodegeneration that leads to aberrant behaviour and loss of memory functions. Mostly, a reduced cholinergic transmission in cortical and hippocampus neurons has been found as the leading cause to cognitive impairment, which manifests as dementia, amnesia, forgetfulness, and the development of AD [4–6]. Epidemiological research claims that people with type 2 diabetes mellitus have a 20–70% increased risk of acquiring AD when compared to people without type 2 diabetes [7]. A variety of factors have been associated to the pathogenesis of diabetes associated AD, including brain insulin sensitivity or resistance, changes in glucose control, generation of reactive oxidative species, mitochondrial dysfunction, pro-inflammatory cytokines with glycation end products [8–10]. However, the exact pathophysiology of AD is still unclear which limits development of any specific medication to treat the disease.

Currently, the USFDA has only authorized a handful number of medications for the treatment of AD, including donepezil, galantamine, memantine, rivastigmine, and lecanemab [11]. Majority of these medications target the N-methyl-D-aspartic acid receptor, amyloid beta (Aβ), or acetycholineesterase (AChE), providing symptomatic relief rather than addressing the fundamental cause of the disease [12, 13]. The sole etiological therapy for AD among all the medications authorized by the USFDA is aducanumab, however dissatisfied phase III study results restricted its commercialization [14, 15]. At present, the majority of marketed drugs prescribed for AD frequently cause side effects including hepatotoxicity, diarrhoea, or sleeplessness after prolonged usage [16]. Since AD is a chronic disorder, patients who are diagnosed with it are required to take the recommended medicine for the rest of their lives, which eventually results in mild to severe unpredictable symptoms. Coupled to this, the conventional delivery challenges across blood–brain barrier (BBB) make the treatment further complex [17]. Thus, to enhance the quality of life in AD patients, efficacious, compatible, and cost-effective therapeutic agents with minimal side effects are present need of hour.

Over the past years, nanocarrier mediated drug targeting has been evolved as an efficient tool by the formulation experts to address challenging aliments including neurodegenerative disorders [18, 19]. As, it is well acknowledged that the discovery/development of new drug is exceedingly arduous, time-consuming, and costly, new-age scientific pool has switched their focus towards novel delivery approaches for approved medications having established memory enhancement functions to target AD.

**Fig. 1:**
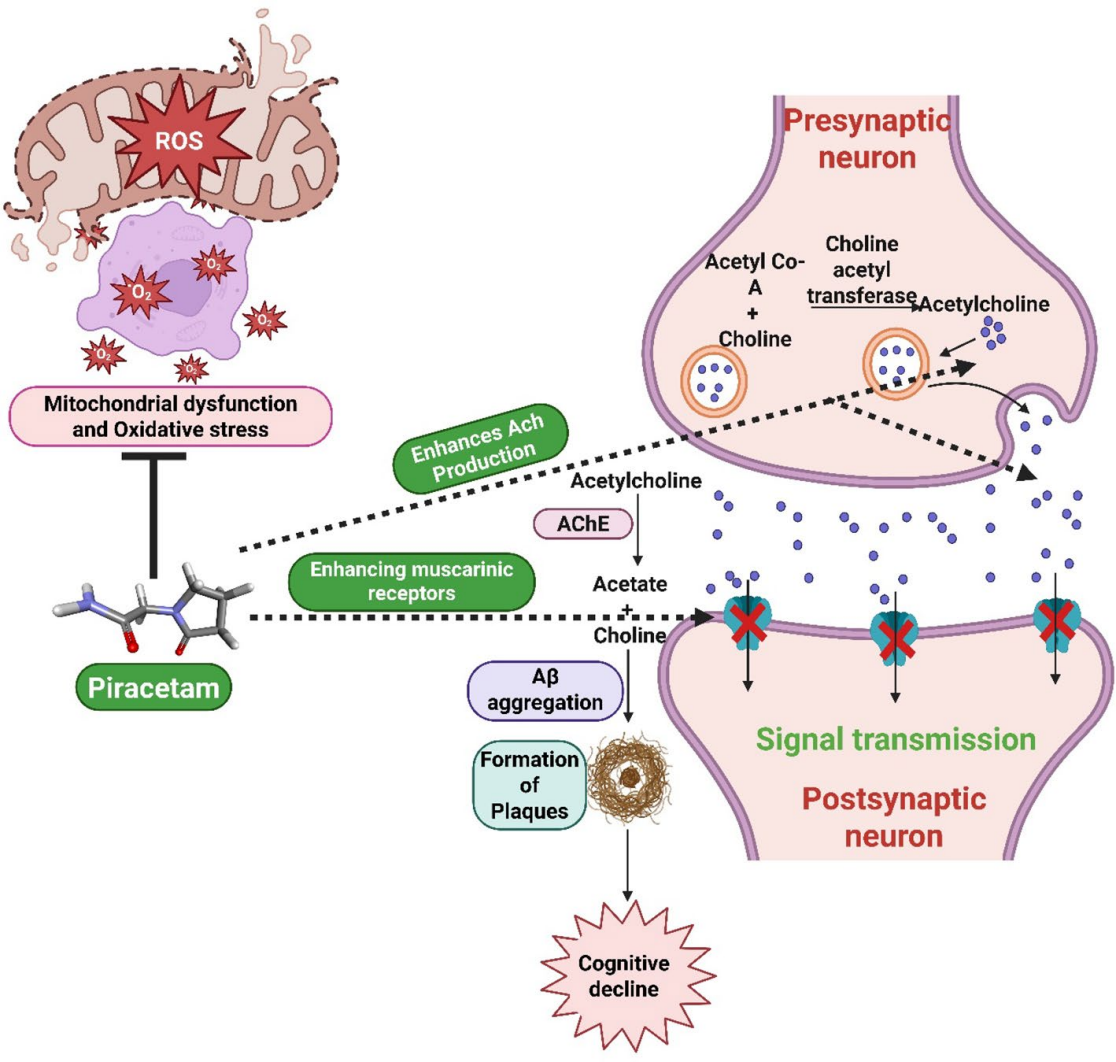
Mechanistic insights into the action of piracetam in modulating cholinergic transmission and mitochondrial dysfunction in cognitive dysfunction

Piracetam is a cyclic derivative of γ-aminobutyric acid (one of the first known synthetic nootropics), which improves cognitive performance by enhancing hippocampal acetylcholine (ACh) levels and increasing the number of muscarinic cholinergic receptors in the frontal cortex [20, 21]. The action of piracetam on its cognitive-enhancing effects is due to a combination of many other complementary mechanisms, other than a single binding site. Moreover, piracetam interacts with cellular and mitochondrial membranes, modifying their fluidity and functionality, hence offering neuroprotection against neurodegenerative diseases resulting from mitochondrial malfunction and oxidative stress [22]. All the actions, individually and in combination, reduce synaptic connectivity and cognitive functioning. However, delivery of piracetam in a conventional dosage form does not ensure its sufficient BBB permeation and optimal therapeutic concentration at the brain tissue [23]. Thus, innovative delivery strategies are being explored for efficient brain delivery of piracetam to elicit the intended therapeutic outcome with reduced side effects. The proposed mechanism of action of piracetam in modulating cognitive dysfunction (Fig. 1).

Solid lipid nanoparticles (SLNs) have recently sparked interest as an attractive nanomodality owing to their high stability, drug loading capacity, high lipophilicity and easily tuneable surface characteristics, which make them ideal candidate for encapsulating different potent drugs to achieve desirable therapeutic efficacy [24]. In a recent research, SLNs containing memantine hydrochloride and tramiprosate was developed as nose-to-brain delivery system for AD. The SLNs had particle size of 159.9 ± 0.569 nm, entrapment efficiency of 99.24% (memantine hydrochloride) and 89.99% (tramiprosate), with sustained release for 48 h. *In vivo* studies revealed a fourfold increase in brain concentration (memantine hydrochloride: 177.96 ± 18.37 µg/mL; tramiprosate: 30.29 ± 2.01 µg/mL) over the pure drugs [25]. Asiatic acid-loaded SLNs was reported elsewhere for treatment of AD. A higher entrapment efficiency and yield percentage was reported for the experimental SLNs. Ex vivo studies depicted significantly higher permeation of asiatic acid through olfactory mucosa (49.63 ± 11.03 µg/cm^2^ in 6 h). *In vivo* study exhibited significantly higher brain accumulation of asiatic acid delivered through SLNs after 8 h as compared to intravenous administration of free drug (*p* < 0.05) [26]. Curcumin-loaded SLNs in a transgenic AD mouse model showed significant neuroprotective effects. The SLNs (150 mg/kg) improved memory retention as compared to free curcumin (***p < 0.001), by elevated Cyclin-D1 and Bcl-2 expression, and reduced caspase-3 cleavage [27].

Several recent research have evidenced potent neuroprotective action of piracetam in seizures, dementia, and cognitive impairment in various animal models. Piracetam-shatavarin IV nanoemulsion showed strong binding affinities to key AD targets viz*.* GSK-3β (− 8.82 kcal/mol), amyloid-β (− 6.83 kcal/mol) as depicted from molecular docking analysis. A synergistic combination index of 0.10843 at a 1:1 ratio indicated enhanced antioxidant activity and sufficient brain delivery [28]. In another research, piracetam-loaded magnetic chitosan nanoparticles significantly mitigated thiacloprid-induced neurotoxicity *in vivo* [22]. Piracetam loaded nanoparticles restored short-term memory in AD rats by increasing spontaneous alternation by 89.1%, elevated AChE activity by 145.5%, and boosted Glutathione (GSH) levels by 94.5%, while reducing lipid peroxidation by 36.5% as compared to control and plain piracetam.

Piracetam (200–400 mg/kg) significantly reversed doxorubicin-induced cognitive deficits in rats as reported by mani et al. 2022 [29]. Data showed a sharp reduction in AChE levels from 18.04 to 7.80 ng/mg, TNF-α from 265.6 to 168.8 pg/mg, and Malondialdehyde (MDA) from 33.16 to 26.52 ng/mg, while GSH rose from 0.503 to 0.813 ng/mg in AD rats following piracetam administration. Anti-apoptotic action of piracetam against LPS-induced cellular death was also demonstrated [30].

The current work attempts to fabricate SLNs encapsulating piracetam and to test the *in vivo* efficacy in diabetes induced AD model. Following the development, experimental piracetam loaded SLNs (PSLNs) were characterised by drug loading, size analysis, zeta potential, surface morphology, surface texture and drug release study. Various neurobehavioral, biochemical, and *in vivo* antioxidant activities of PSLNs were assessed and compared to plain piracetam. The brains of the streptozotocin (STZ)-induced diabetic rats were also examined for AChE, nitrite and Aβ1-42 content.

## Experimentals

### Materials

Piracetam, Polylactic-co-glycolic acid (PLGA 85:15) and STZ were acquired from Sigma-Aldrich Co., St Louis, MO, USA. Hi media (Hyderabad, India) provided the polyvinyl alcohol (PVA), Soy lecithin (SL) 3-(4,5-dimethylthiazol-2-yl)-2,5-diphenyl-2H-tetrazolium bromide (MTT), Dulbecco’s Modified Eagle Medium (DMEM), ascorbic acid, 2,2-Diphenyl-1-Picrylhydrazyl (DPPH), and 2, 2′-azino-bis 3-ethylbenzthiazoline-6-sulfonic acid (ABTS). Ethylenediamine tetra acetic acid (EDTA), pyrogallol, thiobarbituric acid, hydrochloric acid, 5,5’-dithiobis (2-nitrobenzoic acid) (DTNB) and trichloroacetic acid (TCA) were bought from Merck, Hyderabad, India. The source of the other chemicals was SRL Pvt. Ltd. (Mumbai, India).

#### Cell line

HT-22 mice neuronal hippocampus cell line (SCC129) was collected from the Salk Institute, La Jolla, CA, USA. CTX TNA2 astrocyte, type I cell line (CRL-2006) was collected from American Type Culture Collection, Manassas, VA.

#### Animals

For the experiment, a total of 78 Albino rats (3-month-old, both sexes) weighing 185–200 g were used. The animals were pre-acclimatised in the Central Animal House Facility, School of Pharmaceutical Sciences, Siksha ‘O’ Anusandhan University, Bhubaneswar, India. A total of 30 rats were used for behavioural, antioxidant, biochemical and histopathological studies, whereas 48 rats were used for the brain pharmacokinetic study. The rats were separately housed in polypropylene cages according to proper protocol and supplied normal animal diet and drinking water. The rats were maintained in controlled environmental conditions during the entire period of the study that include a temperature of around 25 ± 5 °C, a humidity level of 25–35%, and a 12 h light–dark cycle. Rats were acclimatised for 14 days before commencement of the study. The experimental procedure was approved by the Institutional Animal Ethical Committee (approval number: IAEC/SPS/SOA/216/2025). Animal care regulations were strictly monitored during using and caring of laboratory animals.

### Formulation and physicochemical characterization

#### Method of formulation of piracetam loaded solid lipid nanoparticles

A multiple-emulsion solvent evaporation technique was used to fabricate experimental PSLNs, with certain adjustments made to previously published report [31, 32]. Briefly, measured amount of piracetam, PLGA, and SL were dissolved in 3 mL of dichloromethane and 2 mL of methanol (as organic phase). PVA (2% w/v) dispersion was used as the aqueous phase. Using a high-speed homogenizer (IKA Laboratory Equipment, Model T10B Ultras-Turrax, Staufen, Germany), 1.5 mL of aqueous phase was mixed dropwise into organic phase at an optimal speed of 22,500 rpm. The produced primary emulsion was then gradually mixed with 40 mL of 1% (w/v) PVA solution while continuously homogenizing (22,500 rpm), yielding a secondary emulsion. Following this, the secondary emulsion was put on a magnetic stirrer and swirled 12 h to evaporate the organic solvent with precipitation of SLNs. The resultant SLNs underwent a 45-min centrifugation at 15,000 rpm. Following separation, the supernatant was discarded and the pellets were reconstituted in Milli-Q water (Millipore Corp., Billerica, MA, USA) and centrifuged to eliminate any remaining PVA. The process was repeated for three times to ensure complete removal of the adherent free polymer from SLNs surface. Resultant SLNs was lyophilized for 10 h using a Lyophilizer (Laboratory lyophilizer; IIC Industrial Corporation, Kolkata, India) following a pre freezing at − 40 °C. A cryoprotectant of 5% w/v mannitol was used during the lyophilization cycle.

#### Fourier transform infrared spectroscopy (FTIR)

To predict the compatibility and any potential interactions in between piracetam, and selected excipients, FTIR was employed used both prior to and following the formulation development. Briefly, PLGA, PVA, SL, piracetam, their physical mixture and the prepared lyophilized PSLNs were subjected to FTIR analysis at room temperature using a Nicolet Instruments, Madison, Wisconsin, USA (Magna-IR 750, Series II analyzer) [33, 34]. During analysis, a scan range of 4000–600 cm^−1^ was set with spectral resolution at 4 cm^−1^. Characteristic peaks of the components were then analyzed using the spectra manager software (version 2.0).

#### Differential scanning calorimetry (DSC)

To assess any chemical interaction or instability in the formulation, DSC has been employed as an inventible tool. Briefly, weighed amounts of pure piracetam, and PSLNs were evaluated using a DSC (Mettler Toledo DSC 1, Switzerland) at a heating rate of 10 °C per minute between 30 and 300 °C [35]. All experiments were carried out in an inert nitrogen atmosphere (50 ml per minute).

#### X-ray diffraction analysis (XRD)

An X-ray diffractometer (D8 Discover, Bruker, Germany) was used to investigate the drug’s crystallinity/amorphization in the formulation [36]. To ascertain the impact of encapsulation on its crystalline structure, the XRD patterns of the PSLNs were contrasted with those of pure piracetam. The analysis was carried out under scattered radiation data utilizing Cu as the anode material with an anode voltage of 40 kv and a current of 15 mA. CuKα radiation was applied to each sample at a scan speed of 1° min^−1^, and measurements were conducted at varied scanning angles between 5 to 70°.

#### Yield percentage

Lyophilized PSLNs were weighed and % yield was computed as per the following formula [37].

$$ \% {\mkern 1mu} of{\mkern 1mu} yield = \left( {\frac{{Quantity\,of\,{\mathrm{PSLNs}}\,obtained\,after\,lyophilization}}{{Total\,quantity\,of\,ingradients\,utilized\,in\,each\,batch\,formulation}}} \right) \times 100 $$

#### Percentage piracetam loading and loading efficiency

In an Eppendorf tube, 2 mg of lyophilized PSLNs was dispersed in 4:6 ethanol to water combination [38]. The dispersion was vortexed (5–7 min), centrifuged (22,500 rpm) and absorbance of the supernatant was measured at 208 nm (UV/VIS spectrophotometer, Shimadzu, Kalbadevi, Mumbai, INDIA).

$$ \% {\mkern 1mu} \,piracetam{\mkern 1mu} \,loading = \left( {\frac{{Piracetam\,content\,in\,{\mathrm{PSLNs}}}}{{Total\,amount\,of\,{\mathrm{PSLNs}}\,acquired}}} \right) \times 100 $$

$$ \% \,{\mkern 1mu} loading\,{\mkern 1mu} efficiency = \left( {\frac{{Practical\,{\mathrm{PSLNs}}\,loading}}{{Theoretical\,{\mathrm{PSLNs}}\,loading}}} \right) \times 100 $$

#### Determination of zeta potential, polydispersity index (PDI) and size distribution

The experimental PSLNs’s zeta potential, PDI and average hydrodynamic particle size (Z-avg) were assessed using a Zetasizer (DLS-nano ZS, Malvern Instrument Ltd, Malvern, UK) [39]. 1 mg of lyophilized PSLNs was redispersed in millipore water, sonicated (2–3 min) and was taken inside the cuvette. The analysis was done at 90° scattering detector angle at 25 °C employing ZEN1002 dip cell with MPT-2 Titrator (pH 1–14). The data were interpreted by the instrument software (DTS software version 4.0).

#### Scanning electron microscopy (SEM)

Scanning electron microscopy (SEM) was used to analyze the experimental PSLNs’ surface morphology and shape (JEOL JSM-6480LV, Japan). Briefly, lyophilized PSLNs was sprinkled on one side of the double adhesive stub followed by gold coating (Fine coat, ion sputter JFC-1110) [40]. PSLNs was analysed using SEM equipped with a backscattered electron detector for imaging. Imaging was carried out using a 15 kV accelerating voltage under high vacuum at various magnifications.

#### Atomic force microscopy (AFM)

The nanometer-scale surface characterisation is provided by the AFM. Briefly, the lyophilized PSLNs was redispersed in milli-Q water, vortexed (2 min), sonicated (3–5 min) and was placed on coverslips [41]. The air dried PSLNs as then analysed using AFM (5500 Agilent Technologies, USA) in tapping mode.

### *In vitro* activity studies

#### *In vitro* release of encapsulated drug from PSLNs

A dialysis method (Himedia dialysis membrane-50, MW 12000 to 14000 Da, Mumbai, India) was employed to depict release of piracetam from SLNs under controlled laboratory condition. Briefly, 5 mg of PSLNs was redispersed in Phosphate buffered saline (PBS), pH 7.4 [42, 43]. The dialysis bag containing the formulation was then immersed inside a beaker containing the PBS and was placed on a magnetic stirrer operated at 300 rpm. 1 ml of the sample at prefixed intervals was withdrawn from the beaker containing release medium with simultaneous replenishment of fresh PBS. Samples were analysed using a HPLC System (Agilent 1260 Infinity II Prime Liquid Chromatography System, New Delhi, INDIA).

#### Estimation of release kinetics

Release kinetics helps to predict mechanism of drug release from the formulation. For the study, the drug release data was fitted to different mathematical kinetic models, viz., Higuchi, first order, zero order, Hixson-Crowell, Korsmeyer-Peppas, Weibull model, etc. [44]. From the coefficient of determination (R^2^), the best fit model was predicted.

#### Stability studies

The stability of experimental PSLNs was assessed using a stability chamber (Inlab Equipment’s, India). Various critical formulation parameters like zeta potential, average particle size, and percentage loading efficiency of PSLNs were compared under different storage conditions. In screw-capped amber-tinted glass vials, the PSLNs suspensions were kept for 90 days at three different temperatures: 2–8 °C, 25 ± 2 °C/65 ± 5% RH, and 40 ± 2 °C/75 ± 5% RH [45]. At predetermined intervals (0, 30, 60, and 90 days), the samples’ average particle size, zeta potential, and loading efficiency were examined. A comparison is made between the outcomes of these parameters prior to and following storage.

#### DPPH scavenging assay

PSLNs’ antioxidant activity was measured using DPPH free radicals scavenging assay. Briefly piracetam and PSLNs were dispersed into 3 mL of 0.1 mM methanolic DPPH solution at various concentrations ranging from 10–50 μg/mL. The solution was allowed to incubate at room temperature for 30 min (under dark conditions). The sample’s absorbance was measured at 515 nm in comparison to methanolic DPPH (as blank) and ascorbic acid (as standard) [46]. The antioxidant effectiveness was expressed in terms of IC_50_ value. The % inhibition was calculated using the following formula

$$ \% {\mkern 1mu} \,of{\mkern 1mu} \,inhibition = \frac{{\left( {absorbance\,of\,control - absorbance\,of\,the\,sample} \right)}}{{absorbance\,of\,control}} \times 100 $$

#### ABTS scavenging assay

To perform the ABTS radical scavenging assay, an equal mixture of 7 mM ABTS and 2.45 mM potassium persulphate was mixed, and the mixture was then incubated for 12–16 h at 25 °C. 1 mL of ABTS stock solution was diluted until the diluted solution’s absorbance reached at 0.7 ± 0.05 (at λ_max_ 730 nm). Piracetam and PSLNs at varying concentrations (10–50 μg/mL) were combined with 1 mL of diluted ABTS solution, and the volume was adjusted to 5 mL using double distilled water [47]. Ascorbic acid was used as standard. Following a 30 min of incubation, the test solutions were compared to the blank solution at a wavelength of 730 nm. The IC_50_ value and percentage of inhibition were calculated using the following formula

$$ \% \,{\mkern 1mu} of\,{\mkern 1mu} inhibition = \frac{{\left( {absorbance\,of\,control - absorbance\,of\,the\,sample} \right)}}{{absorbance\,of\,control}} \times 100 $$

#### Assessment of cytotoxicity of PSLNs in vitro

The MTT test was employed to assess in vitro cytotoxic effect of PSLNs on CTX TNA2 astrocyte, type I cell line, and HT22 mice neuronal hippocampus cell line (SCC129) respectively. Briefly, both the cell lines (1 × 10^5^ cells/ well) were cultured in DMEM supplemented with 10% fetal bovine serum (v/v) in 96-well culture plates and incubated in a CO_2_ humidified incubator (37 ºC for 24 h) [48, 49]. Cells were treated with different dilutions of PSLNs (1, 5, 10, 20, 30, 40 and 50 μg/ml). Control cells were treated with a blank DMEM. Following 24 h of incubation, culture media was discarded and replaced with fresh culture media (100 µl/well) and MTT solution. The culture plates were incubated for another 4–6 h at 37 ºC. The supernatant was removed with the addition of sterile DMSO to each well (100 µl). Following a short incubation period of 2 h, the sample’s absorbance (at 570 nm) was measured.

$$ Percentage{\mkern 1mu} \,cell\,{\mkern 1mu} viability = \left( {\frac{{Absorbance\,of\,treated\,cell\,suspension\,at\,570\,nm}}{{Absorbance\,of\,untreated\,cell\,suspension\,at\,570\,nm}}} \right) \times 100 $$

### *In vivo* neurobehavioral studies

#### Experimental design

For 21 days, animals in Group 1 (control group, n = 6) received 10 mL/kg p.o. of normal saline. STZ (45 mg/kg, i.p.) diluted in a freshly prepared citrate buffer solution (pH 4.4) was administered once to selected number of animals. Post 72 h STZ injection, the animal’s blood glucose levels were measured. After 7 days of STZ induction [50] those animals showing blood glucose levels above 250 mg/dL were divided into four groups (Groups 2 to group 5) of 6 animals each and received saline 10 mg/kg p.o., plain piracetam 100 mg/kg, i.p., PSLNs at 30 mg/kg i.p. and PSLNs 50 mg/kg, i.p., respectively, once daily from day 8 of STZ induction to day 21.

Group-1 (n = 6): Saline (10 ml/kg p.o.) administered control group (21 days).

Group-2 (n = 6): STZ (45 mg/kg i.p.) + Saline 10 ml/kg p.o. (14 days).

Group-3 (n = 6): STZ (45 mg/kg i.p.) + Piracetam (100 mg/kg i.p.) (14 days).

Group-4 (n = 6): STZ (45 mg/kg i.p.) + PSLN-2 (30 mg/kg i.p.) (14 days).

Group-5 (n = 6): STZ (45 mg/kg i.p.) + PSLN-2 (50 mg/kg i.p.) (14 days).

Neurobehavioral investigations were carried out on the 0th day (before the administration of any medication), as well as on the 7^th^, 14^th^, and 21^st^ days (after the administration of the drugs). At the end of the study, the animals were humanely euthanized on the 22nd day (n = 6). Humane endpoints were predefined and included ≥ 20% loss of body weight, persistent anorexia or dehydration, marked behavioural abnormalities (such as lethargy, impaired locomotion, or abnormal posture), or clinical signs of severe pain or distress. The brains were separated and kept for histological examination (n = 3) and to prepare the brain homogenate (n = 3). The brain homogenate was utilized to estimate the AChE activity, nitrite activity, Aβ_1–42_, and *in vivo* antioxidant studies.

#### Y-maze

Y-maze is the preliminary behavioural study associated with memory function of rat. It calculates the proportion of spontaneous alternation behaviour in rats. The Y-maze has typically three arms A, B, and C, each of which is 50 cm long, 10 cm broad, and 20 cm height [51, 52]. The rats were kept at the middle section of the labyrinth and given five minutes to explore the entire area. When the rat entered its four paws into the maze’s arm, a corrected arm entry was recorded. The term “arm alternation” refers to the repeated counting of triplet combination patterns, such as ABC, ACB, BAC, etc., that are shown when the rat enters the arm. The percentage SAB was then calculated using the following formula

$$ \% \;{{Spontaneous}}\,alternation{\mkern 1mu} \,behaviour\; = \;\left[ {\left( {No.\,of\,alteration} \right)/\left( {Total\,arm\,entries - 2} \right)} \right] \times 100 $$

#### Elevated plus maze

The elevated plus maze consists of two open and closed arms having 50 × 10 cm length, which are positioned opposite one another, rising approximately 40 cm above the floor. During the experiment, the experimental rats were faced towards open arms and were allowed to roam throughout the maze. The transfer latency was calculated by measuring the entry of rats into the closed arms with all four paws, which was recorded for every rat [52]. A cut-off time of 60 s was fixed for each animal movement. After 24 h, the retention transfer latency was measured again.

#### Radial arm maze

Another reliable neurobehavioral test for assessing a rat’s memory function is the radial arm maze, which counts the number of correct responses [53]. For the experiment, rats were placed in the middle of the maze and were given free access to roam across the maze until they reached the end of each radiating arm. Re-entry into the same arm was regarded as a memory dysfunction, but entrance into the non-repeated arms is recorded as valid entry. The number of correct responses was recorded for eight minutes.

#### Morris water maze

The Morris water maze is a widely recognized tool for assessing hippocampus-dependent spatial learning, memory, and spontaneous motor function. In this study, rats from each experimental group were first placed one by one on the maze platform, which was situated just below the water’s surface, for 30 s. Subsequently, the animal was released into the pool and was given 60 s to locate into the merged platform. If it failed to do so within the allotted time, additional 10 s time was given to locate into the merged platform. Each animal underwent four trials per day, spaced 10 min apart, to evaluate learning acquisition [54]. During these trials, the animal was introduced into different quadrants of the maze in a randomized order. For memory retention assessment, the platform was concealed by rendering the water opaque. Animals were tested on days 0, 7, 14, and 21 by placing them into a selected quadrant, and their spatial memory retention was evaluated. The time taken by the rat to locate into the merged platform is referred as escape latency.

### *In vivo* antioxidant and biochemical studies

#### Preparation of brain homogenate

Following the neurobehavioral assessment, the animals were fasted for 12 h before being anesthetized with a solution comprising xylazine (12.5 mg/kg i.p.) and ketamine (87.5 mg/kg i.p.). The brains were then isolated and washed with ice cold water. A high-speed homogenizer was used to homogenize the whole brain at 1800 rpm in addition to 0.3 M phosphate buffer (pH = 7.4). A cooling centrifuge was then used to centrifuge those homogenates for 30 min at 15,000 rpm at 4 °C [55]. After centrifugation, the supernatant was stored at − 80 °C for further biochemical and antioxidant analyses.

#### Estimation of superoxide dismutase (SOD)

The pyrogallol approach was utilized to test the SOD content using a UV–visible spectrophotometer (UV/VIS spectrophotometer, Shimadzu, Kalbadevi, Mumbai, INDIA). A blank solution consisting of 1 mM (0.5 ml) EDTA, 0.05 mM (1.5 ml) tris buffer, and 0.2 mM (1 ml) pyrogallol was used to test SOD activity [56]. The test solution was created by adding 50 µl of brain homogenate supernatant to the blank solution. Pyrogallol’s auto-oxidation was carried out in the dark. The absorbance of the test sample was measured at 420 nm and was compared to that of blank solution.

The percentage of inhibition was calculated using,

$$ \user2{\%\, Inhibition} = Abs\left( {control} \right) - Abs \left( {sample} \right)/Abs \left( {control} \right) \times 100 $$

SOD content was represented as mmol/mg protein and was computed by placing each percentage of inhibition in the standard curve.

#### Estimation of GSH

The reaction of Ellman’s reagent (DTNB) with thiol groups was used to calculate the GSH concentration. For the experiment, 100 µl of brain homogenate supernatant, 0.02 M EDTA, and an equivalent quantity of 10% TCA were used [56]. Following centrifugation for 10 min at 4 °C and 2000 rpm, the supernatant was collected. To this 2 ml of PBS (pH 8.4), 0.4 ml of double-distilled water and 0.05 M DTNB was added. Upon gentle shaking, the colour of the mixture gradually shifted to pale yellow. The absorbance of the mixture was measured at 412 nm within 15 min, and the GSH content was reported as µg/mg of protein.

#### Estimation of catalase

The catalase enzyme activity was measured using a UV visible spectrophotometer (UV/VIS spectrophotometer, Shimadzu, Kalbadevi, Mumbai, INDIA) by measuring the hydrogen peroxide (H_2_O_2_) oxidation capacity at 240 nm. The blank solution was made by adding 2 ml of PBS (0.05 M, pH = 7.4) and 1 ml of H_2_O_2_ (0.019 M) [57]. The test solution was made by adding 50 µl of brain homogenate to the blank solution. At 240 nm, the quantity of H_2_O_2_ that was broken down was measured. The amount of catalase was calculated as nmol H_2_O_2_ degraded/min/mg protein.

#### Estimation of lipid peroxidation

Membrane lipid peroxidation is indicated by MDA. It is identified spectroscopically by the presence of thiobarbituric acid-malondialdehyde. About 2 ml of 15% TCA, 2 ml of 0.37% thiobarbituric acid, and 2 ml of 0.35 N HCl made up the blank solution [58]. To prepare each test sample group, 0.1 ml of homogenized brain supernatant was added to the blank solution. The samples were then analysed using an UV visible spectrophotometer (UV/VIS spectrophotometer, Shimadzu, Kalbadevi, Mumbai, INDIA) at 532 nm. MDA content was computed using the malonaldehyde bis (dimethyl acetal) standard curve and reported as nmol/mg of protein.

#### Estimation of nitrite

The Griess reagent (500 μl) was used to detect the amounts of nitrite, a marker of nitrosative stress in the brain. The reagent contained a 1:1 combination of 1% sulphanilamide in 5% phosphoric acid and 0.1% napthylamine diamine dihydrochloric acid in water [59]. The mixture was incubated for 5 min at room temperature and 0.1 ml of the homogenized brain supernatant was added to it. The absorbance of the sample was measured at 540 nm. The nitrite concentration (µmol/mg protein) was extrapolated from the standard curve of sodium nitrite.

#### AChE activity estimation

For the test blank solution of AChE was made with 0.1 ml of acetylcholine iodide (0.075 M) and 3 ml of PBS (0.01 M, pH 8) and was incubated for 15 min. After adding 0.1 ml of DTNB (10 mM), the mixture was incubated for a further 5 min at room temperature. The test solution was made by mixing the blank solution with 50 μl of the brain homogenate supernatant [60]. The final product of ACh breakdown, generated a yellow colour product (thiocholine) following its combination with dithiol bis nitrobenzoate ions. From thiocholine levels, AChE activity in the brain was estimated. A UV visible spectrophotometer (UV/VIS spectrophotometer, Shimadzu, Kalbadevi, Mumbai, INDIA) was used to detect the absorbance of thiocholine at 412 nm. The percentage of inhibition was then calculated using the following formula.

$$ \user2{\%\, Inhibition} = Abs\left( {control} \right) - Abs \left( {sample} \right)/Abs (control \times 100 $$

#### Aβ estimation

Aβ_1-42_ levels in the brain homogenate were measured using the rat Aβ_1-42_ ELISA kit (Elabscience, Catalog No: E-EL-R1402). The standard solution was initially created using the serial dilution method in order to obtain a standard calibration curve. An Aβ_1-42_ immunoassay was performed using a microtiter plate reader and the solid-phase sandwich method of ELISA [61]. Following the addition of 50 μL of substrate reagent, the ELISA plate was incubated for 15 min at 37 °C. The reaction mixture was terminated by adding 40 μL of stop solution. At 450 nm, the blue colour changed to yellow. The relevant standard curve was then used to calculate the protein.

### Histopathological studies

In order to examine histological parameters, the brain tissues (n = 3) were extracted and stored in a 10% formalin solution. Specified percentage of ethanol, water, and xylene-based solutions were then used to fix, dry, and clean the tissues. The tissues were then sliced into thin Sects. (5–7 μm) thick and were preserved in paraffin wax. Lastly, a solution of haematoxylin and eosin dye was used to stain the tissues [62]. The tissues were observed under a trinocular biological microscope.

### Brain pharmacokinetic study

For quantitative determination of amount of piracetam concentrated in experimental rat brain, following i.p. administration, a brain pharmacokinetic investigation was performed in rats with diabetes induced cognitive dysfunction (2 groups, total 48 animals). For pharmacokinetic study, one group was treated with plain Piracetam (100 mg/kg i.p. single dose) and the other with PSLN-2 (30 mg/kg i.p. single dose). Brain samples were obtained following sacrifice of the animals at specified intervals such as 0.5, 1, 2, 4, 6, 8, 12, and 24 h post injection. Brain of each rat was isolated, homogenized (using PBS, pH 7.4) followed by centrifugation at 4000 rpm for 15 min. The supernatant of brain homogenate was collected and preserved at 4 °C till further analysis. Quantification of piracetam in brain tissue was conducted using a liquid chromatography-tandem mass spectrometry (LC–MS/MS) technique [63].

For sample preparation, about 100 µL of the supernatant was combined with 5% trichloroacetic acid (for protein precipitation). About 50 µL of oxiracetam was added to each sample (as internal standard). Samples were vortexed (10 min) and then centrifuged at 4000 rpm (5 min). A 100 µL aliquot of the supernatant was diluted with 100 µL of milli Q water, mixed thoroughly. A Zorbax SB-Aq column (150 × 2.1 mm, 3.5 µm) was employed for chromatographic separation using mobile phase as acetonitrile and formic acid (1%) at 10:90 v/v. The flow rate was maintained at 0.3 mL/min with a total duration of 3.0 min. About 20 µL of the prepared sample was injected into the LC–MS/MS column. MS data acquisition was carried out in multiple reaction monitoring mode with a positive electrospray ionization interface. Various Pharmacokinetic parameters, viz. Different pharmacokinetic parameters i.e. area under the curve (AUC), volume of distribution (V_d_), area under the first moment curve (AUMC), mean residence time of the drug (MRT), rate of tissue clearance (Cl_t_), etc. were determined using non-compartmental PK Solver software.

#### Statistical analysis

The results were displayed as mean ± SD. One-way ANOVA followed by Tukey’s post hoc t-test was employed for statistical analysis. The Shapiro–Wilk test was used to assess normality, and the Brown-Forsythe test was used to assess the homogeneity of variance. The test was evaluated using GraphPad Prism (version 5.0). One-way ANOVA was applied when these assumptions were satisfied. Student’s t-test was applied for MTT and pharmacokinetic studies. *p* < 0.05 was considered significant.

## Results

### Formulation development and physicochemical evaluation

#### Formulation development of experimental PSLNs

Experimental PSLNs was developed keeping varying amount of drug, polymer and lipid. We have kept PLGA amount constant in each formulation batch. Several batches of PSLNs were developed, out of which three suitable formulations (viz*.* PSLN-1, PSLN-2, PSLN-3) having desired physicochemical characteristics in terms of yield percentage, average particle size, zeta potential, loading efficiency were selected. The composition and characterization of selected PSLNs is depicted in Table 1.

**Table 1:** Formulation ingredients, % yield, % piracetam loading and % loading efficiency of piracetam loaded solid lipid nanoparticles (PSLNs)

Formulation code	Amount of drug (mg)	Amount of PLGA (mg)	SL (mg)	% Yield (w/w)	% Loading (w/w)	% Loading efficiency (w/w)	Average particle size (d.nm)	Poly dispersity index (PDI)	Zeta potential (mV)
PSLN-1	40	100	100	58.3 ± 1.3%	12.4 ± 1.2%	66.5 ± 1.5%	127.01 ± 2.1	0.8 ± 0.7	− 45.1 ± 1.1
PSLN-2	60	100	50	75.5 ± 1.5%	20.1 ± 0.7%	82.3 ± 2.2%	87.3 ± 1.1	0.2 ± 0.5	− 38.2 ± 0.3
PSLN-3	100	100	30	43.2 ± 0.6%	9.2 ± 0.9%	52.6 ± 0.7%	185.1 ± 1.8	0.5 ± 1.6	− 29.7 ± 1.9

In the reported formulations, amount of piracetam and SL was varied, while keeping the amount of major polymeric excipient PLGA was constant. In each formulation batch, 100 mg of PLGA was used. PSLN-2 was achieved the highest drug loading % among three reported formulations

PSLN: Piracetam loaded solid lipid nanoparticle; PLGA: Polylactic-co-glycolic acid; SL: Soy lecithin. Data show mean ± SD (n = 3)

#### FTIR analysis

**Fig. 2:**
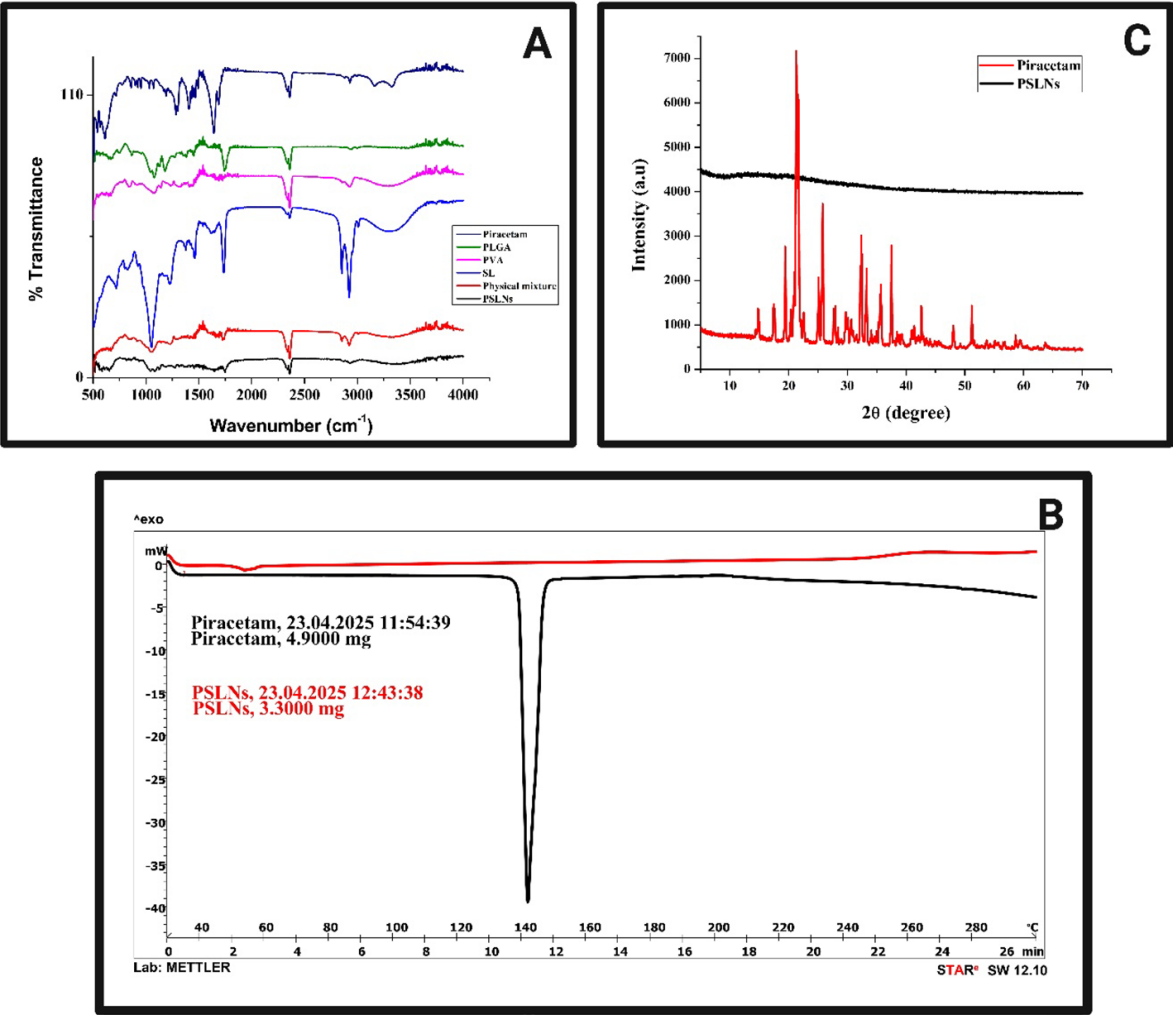
**A** Fourier transform infrared spectroscopy (FTIR) analysis of piracetam, polylactic-co-glycolic acid (PLGA), polyvinyl alcohol (PVA), soy lecithin (SL), physical mixture of piracetam and excipients, and piracetam loaded solid lipid nanoparticles (PSLNs); **B** Differential scanning calorimetry (DSC) thermogram of piracetam, and PSLNs; **C** X-ray diffraction analysis (XRD) of piracetam and PSLNs

FTIR spectra of piracetam, SL, PLGA, PVA, physical mixture and PSLNs showed absence of any major shifting in the characteristics peaks of drug/polymer/lipid in the physical mixture or formulation (Fig. 2A). Characteristic peaks of piracetam were found at 3724.68 cm^−1^ (N–H stretching), 3159.79 cm^−1^ (weak C-H stretching in pyrrolidine ring), 1688.37 cm^−1^ (NH_2_–O–C stretching), 1642.09 cm^−1^ (C = O stretching), 1285.32 cm^−1^ (C–N stretching). For PLGA, the characteristics peaks were observed at 1450.6 cm^−1^ (C-H bending) and 1630.12 cm^−1^ (C = O stretching). For SL, peak at 1735.43 cm^−1^ represented the ketonic group (C = O stretching) and peak at 2923.34 cm^−1^ represented C-H stretching. Similarly for PVA, peaks at 3303.67 cm^−1^ (O–H stretching) and 1412.49 cm^−1^ (C-H bending) was found. The distinctive characteristics peaks of PLGA, PVA, SL and piracetam were observed in the physical combination. In the physical mixture, peak at 1450.6 cm^−1^ (C-H bending) and 1630.12 cm^−1^ (C = O stretching) was observed for PLGA. Similarly, peaks at 3301.54 cm^−1^ (O–H stretching) and 1412.49 cm^−1^ (C-H bending) was detected pertaining to PVA. Peaks at 2922.59 cm^−1^ (C-H stretching) and 1735.55 cm^−1^ (C = O stretching) could be attributed to SL. The peaks at 3724.54 cm^−1^ (NH stretching) and 1691.27 cm^−1^ (NH_2_–O–C stretching) were attributed as the characteristic peaks for piracetam. Similarly, distinctive peaks were observed at 3724.21 cm^−1^, 1642.53 cm^−1^, 1285.87 cm^−1^ in the PSLNs, depicting absence of any significant interaction in between the selected drug and polymers/lipid. However minor shifting in the peak may be attributed to the presence of weak physical interaction like Van der waals, electrostatic, dipole–dipole interaction etc*.* which might beneficial for stable formulation development.

#### DSC analysis

DSC was used to assess the physiochemical state, including any chemical interactions in the formulation as well as crystalline behaviour and degradation profile. The crystalline nature and purity of the drugs are justified by the thermogram’s distinct, sharp single endothermic peak for pure piracetam at 140.31 °C, which corresponds to their melting temperature (Fig. 2B). PSLNs’ DSC curves showed absence of any sharp peaks as compared to pure piracetam. The endothermic fusion peak of piracetam was broadened in PSLNs signifying a change from the crystalline to the amorphous phase following encapsulation of the drug within the nanoparticle core or matrix. Further, absence of any new peaks for PSLNs indicated stable and compatible nature of drug and formulation.

####  XRD analysis

X-ray diffractogram of pure piracetam revealed the characteristic peaks at 19.444°, 21.272°, 21.526°, 25.815°, 32.364°, and 37.490° (2θ) with corresponding interplanar distances of 4.561 Å, 4.1735 Å, 4.1247 Å, 3.4484 Å, 2.7639 Å, 2.3969 Å respectively (Fig. 2C). The crystalline form of piracetam was established by these intense, sharp peaks. Contrary to that, PSLNs showed absence of any sharp peaks. Substantial decrease in peak height and intensity in PSLNs as compared to pure piracetam justified amorphization of piracetam following encapsulation inside the nanoparticle core structure. This would be helpful for improved dissolution of the formulation.

#### Yield percentage, percentage piracetam loading, and loading efficiency

Selected batches of PSLNs had a satisfactory yield percentage. Out of the reported batches, PSLN-2 had the highest yield percentage (75.5 ± 1.5% w/w). The drug loading percentage for each formulation differed, and we observed that PSLN-2 had a larger drug load as 20.1 ± 0.7% (w/w) than the remaining two formulations (PSLN-1 at 12.4 ± 1.2% w/w and PSLN-3 at 9.2 ± 0.9% w/w) (Table 1). Similarly, PSLN-2 loading efficiency (82.3 ± 2.2% w/w) was higher than other two reported formulations. The standardized process parameters may be responsible for the satisfied drug loading of the formulations.

#### Zeta potential, Z-avg. and PDI analysis.

Data indicated that the average particle size (Z-avg.) of the selected formulations were within desired nano size range (Table 1). Out of the three reported batches, PSLN-2 had smaller average size of 87.3 ± 1.17 nm with a low PDI value (0.2 ± 0.5) than other two formulations (PSLN-1: 127.01 ± 2.1 nm, -45.1 ± 1.1; PSLN-3: 185.1 ± 1.8, -29.7 ± 1.9). All reported formulations exhibited negative surface charge. The zeta potential of PSLN-2 was found as -38.2 ± 0.3 mV, suggesting its improved suspension stability.

Based on percentage yield, average particle size, percentage loading, loading efficiency PSLN-2 was selected as the suitable formulation to carry out further studies.

#### SEM

**Fig. 3:**
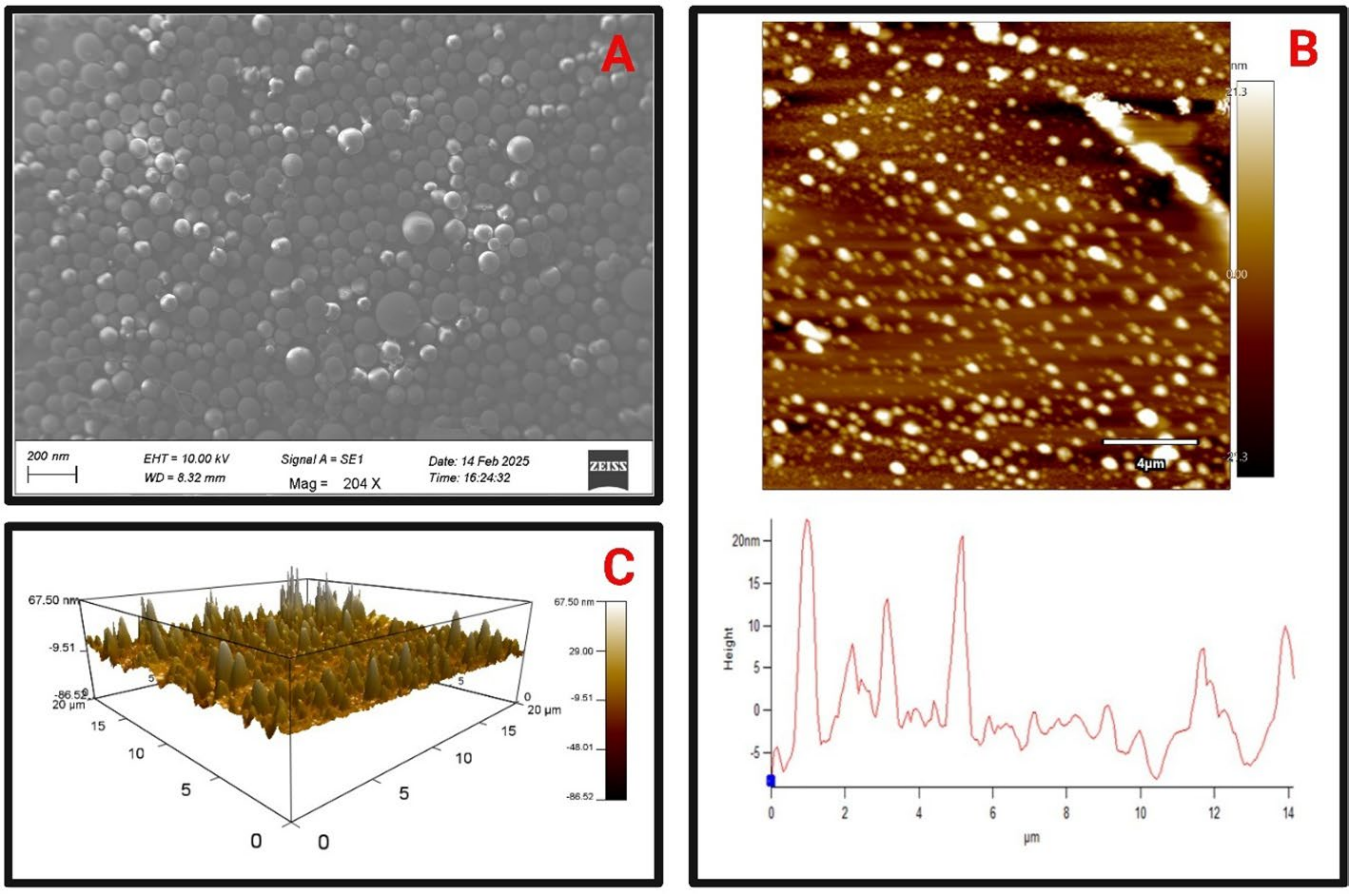
**A** Scanning electron microscopy (SEM) of piracetam loaded solid lipid nanoparticle-2 (PSLN-2) with a magnification of 204 × (Scale 200 nm). All the particles were found spherical with smooth surface and within desired nanorange; **B** Atomic force microscopy (AFM) topographic two-dimensional topographic image of PSLN-2 (top view); **C** AFM forward-scanned three-dimensional topographic image

SEM was used to examine the surface morphology of the PSLN-2. SEM photograph showed smooth surface of PSLN-2 with clear spherical shaped nanostructures (Fig. 3A). Nanoparticles were found having homogenous size distribution pattern within desired nanosize range. Standardized production steps with optimized formulation composition might be assigned for proper formation of experimental PSLN-2.

#### AFM

AFM study demonstrated that the PSLN-2 as perfectly spherical, having a smooth surface, and closely packed arrangement Fig. 3B displays the top view of the AFM topographic two-dimensional (2D) picture of PSLN-2 with a height range of − 21.3 to + 21.3 nm, and Fig. 3C displays the AFM forward topographical three-dimensional (3D) image of the same site with a height range of − 86.52 to + 67.50 nm. Line profile analysis revealed that the average surface height of PSLN-2 was approximately 18.04 nm suggests total film roughness with a discernible PSLN-2. Furthermore, the image depicted uniform, spherically shaped PSLN-2 devoid of any visible pores or crevices. One probable explanation for PSLN-2 smooth surface is the identical stacking of hydrophilic and amphiphilic emulsifiers, specifically SL, across the solid–liquid lipid core surface.

### In vitro studies

#### In vitro release study and estimation of release kinetics

**Fig. 4:**
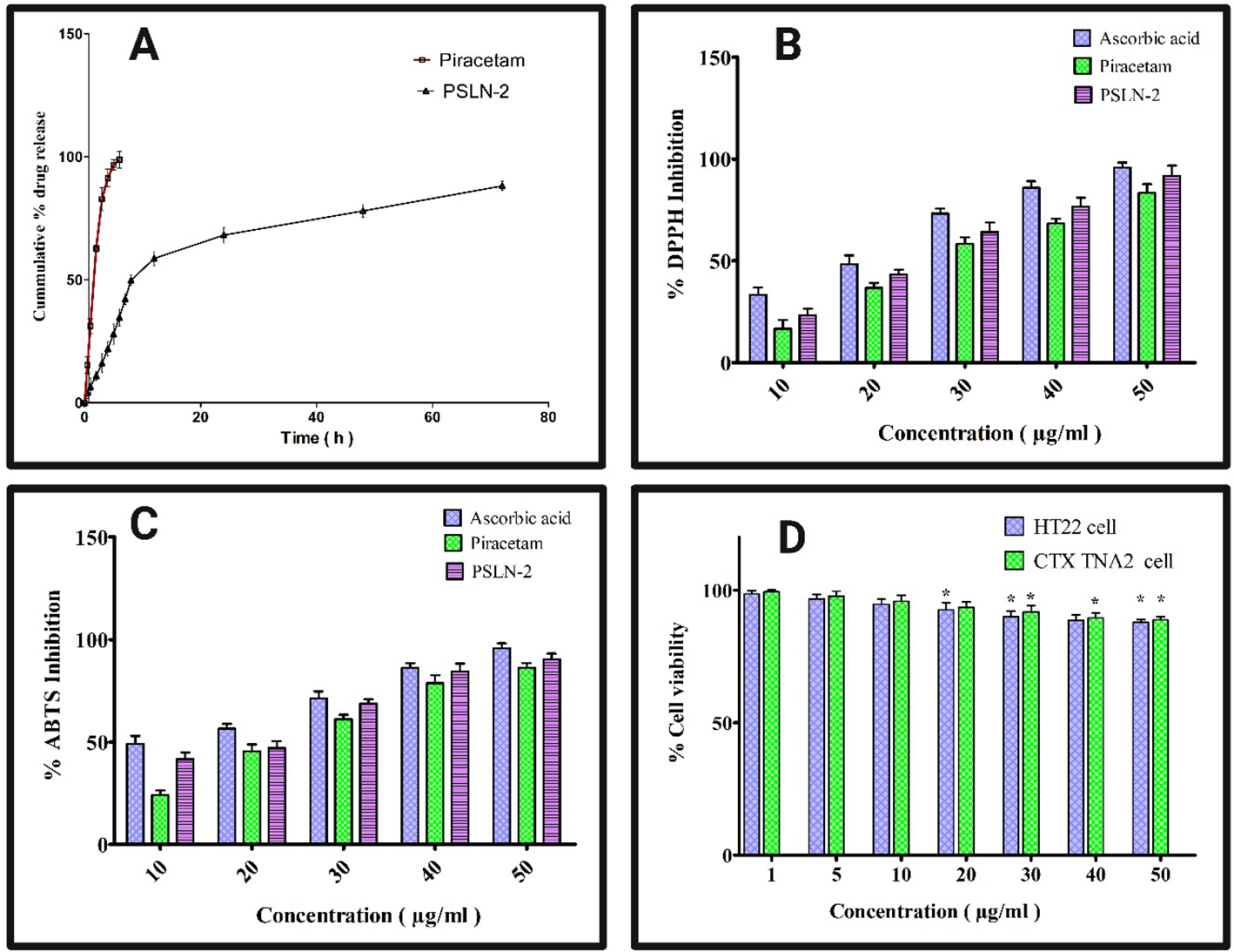
**A** *In vitro* release study of piracetam loaded solid lipid nanoparticle-2 (PSLN-2) in phosphate-buffered saline (PBS), pH 7.4. The study depicted a sustained release profile of piracetam from PSLN-2, whereas the burst release was observed for plain piracetam; **B** Percentage of inhibition of the DPPH scavenging activity by ascorbic acid, piracetam and PSLN-2 (IC_50_:18.92 ± 1.7, 27.8 ± 1.18, and 23.43 ± 0.21 μg/mL); **C** Percentage of inhibition of the ABTS scavenging activity by ascorbic acid, piracetam and PSLN-2 (IC_50_:13.34 ± 1.23, 2 3.9 ± 1.56, and 18.1 ± 1.04 μg/mL). All the data are represented as Mean ± SD where n = 3; **D** *In vitro* cytotoxicity study of PSLN-2 on CTX TNA2 astrocyte (CRL-2006) and HT22 mice neuronal hippocampus cell line (SCC129). Data showed biocompatible nature of experimental PSLN-2 with more than 85% cell viability at the highest tested concentrations. Data represented as Mean ± SD, where n = 3. Student’s t-test *p < 0.05

The rate and extent of drug release from PSLN-2/marketed formulation was tested *in vitro* using the dialysis technique. Data showed consistent drug release pattern from the formulation up to 72 h study period (Fig. 4A). Initially, the drug release expanded with time. But after 12 h, a relatively sustained release profile was observed from PSLN-2. During the study period, a cumulative amount of 88.13 ± 1.8% encapsulated drug was released from PSLN-2. In case of marketed piracetam (Nootropil®), 98.76% drug released within 6 h of study period. Clearly encapsulation of piracetam within solid lipid nanoparticulate core has extended its release substantially than its conventional form. While, estimating the mechanism of drug release from PSLN-2, it was observed that the piracetam release pattern was best fit into Higuchi kinetic model with good linearity (R^2^ = 0.902) (Table 2).

**Table 2:** Estimation of kinetics of drug release from plain piracetam and experimental piracetam loaded solid lipid nanoparticle-2 (PSLN-2)

Sl. no	Release kinetic model	Correlation coefficient of piracetam	Correlation coefficient of formulation (PSLN-2)
1	Zero-order model	0.8918	0.7181
2	First-order model	0.2074	0.0929
3	Higuchi model	0.9608	0.902
4	Hixon-Crowell model	0.6527	0.4252
5	Korsmeyer Peppas model	0.5844	0.3592
6	Weibull model	0.9537	0.8223

#### Stability studies

The results of the stability of PSLN-2 under various storage settings are shown in Table 3. The stability data indicated no major changes in critical physicochemical parameters like physical appearance, particle size, % loading efficiency, and zeta potential in PSLN-2 over the course of three months at recommended ICH storage conditions viz*.* 25 ± 2 °C/65 ± 5%RH, 40 ± 2 °C/75 ± 5%RH and 2–8 °C. However, some minor alterations in particle size and loading efficiency of PSLN-2 were observed at 40 ± 2 °C/75 ± 5%RH during a 90-day storage period, though the data were not significantly varied. However, it may be suggested that the formulation should not be exposed to higher temperature conditions during storage to maintain its shelf life and long-term stability.

**Table 3:** Stability study of piracetam loaded solid lipid nanoparticle-2 (PSLN-2) at different storage conditions, *viz.* 2–8 °C, 25 ± 2 °C/65 ± 5%RH and 40 ± 2 °C/75 ± 5%RH respectively

Stability Condition	Days	Particle size (d.nm)	Poly dispersity index (PDI)	Zeta potential (mV)	% Loading efficiency (w/w)
2–8 °C	0	87.5 ± 1.11	0.2 ± 0.3	-38.31 ± 0.22	82.3 ± 2.1
	30	87.6 ± 1.01	0.21 ± 1.2	-38.45 ± 0.28	82.28 ± 1.3
	60	87.7 ± 1.32	0.21 ± 0.6	-38.31 ± 1.22	82.22 ± 0.7
	90	88.3 ± 1.08	0.22 ± 1.3	-37.23 ± 1.17	82.15 ± 0.2
25 ± 2 °C/65 ± 5%RH	0	87.3 ± 1.17	0.2 ± 0.5	-38.22 ± 0.38	82.3 ± 1.2
	30	87.2 ± 1.23	0.21 ± 1.2	-38.45 ± 0.82	82.26 ± 0.3
	60	88.4 ± 2.11	0.2 ± 2.1	-39.29 ± 1.21	82.19 ± 1.8
	90	89.3 ± 1.82	0.22 ± 0.6	-37.67 ± 0.51	81.92 ± 1.5
40 ± 2 °C/75 ± 5%RH	0	87.3 ± 1.13	0.2 ± 1.7	-38.29 ± 0.56	82.2 ± 1.7
	30	88.2 ± 1.65	0.2 ± 1.2	-37.15 ± 1.25	82.07 ± 1.8
	60	88.94 ± 0.27	0.21 ± 2.2	-39.71 ± 2.6	81.87 ± 2.7
	90	90.82 ± 1.38	0.21 ± 2.5	-39.55 ± 2.37	79.42 ± 1.5

PSLN-2 did not show any significant variation in physical appearances, particle size, zeta potential, PDI and % loading efficiency

RH: Relative humidity; Data show mean ± SD (n = 3)

#### DPPH radical scavenging activity

Piracetam and PSLNs’ ability to scavenge free radicals on DPPH was computed as a percentage of inhibition, as shown in Fig. 4B. Piracetam and PSLN-2 showed a scavenging effect similar to that of standard ascorbic acid. Compared to piracetam, the PSLN-2 had a much greater antioxidant impact. At 50 μg/mL, the ascorbic acid, piracetam, and PSLN-2 inhibition percentages were 95.83 ± 2.37%, 83.33 ± 4.37%, and 91.66 ± 5.12%, respectively. The corresponding IC_50_ values of ascorbic acid/piracetam/PSLN-2 were found as 18.92 ± 1.7, 27.8 ± 1.18, and 23.43 ± 0.21 μg/mL, respectively. The study overall indicated higher antioxidant potential of PSLN-2.

#### ABTS activity

The best way to assess the antioxidant ability of hydrogen-donating antioxidants is to oxidize the ABTS solution with potassium persulphate, which produces an ABTS free radical ion [64]. Following treatment with the PSLN-2, the ABTS solution goes through a reduction reaction, which causes the ABTS solution to become discoloured. The inhibition percentage of free radical with PSLN-2/piracetam was compared that of ascorbic acid. At 50 μg/mL, the ascorbic acid, piracetam, and PSLN-2 inhibition percentages were found as 95.74 ± 2.37%, 86.11 ± 2.41%, and 90.37 ± 2.78% with IC_50_ values of 13.34 ± 1.23, 23.9 ± 1.56, and 18.1 ± 1.04 μg/mL respectively (Fig. 4C). The lower IC_50_ value of PSLN-2 as compared to pure piracetam indicates its stronger antioxidant properties.

#### Evaluation of cytotoxicity of piracetam on brain/neuronal cells (in vitro)

PSLN-2’s cytotoxicity (*in vitro*) was evaluated by MTT assay on both CTX TNA2 astrocyte (CRL-2006) and HT22 mice neuronal hippocampus cell line (SCC129). Overall, data showed no potential toxicity of PSLN-2 at different tested concentrations on both the cell lines (Fig. 4D). At 5 µg/ ml, PSLN-2 did not cause any potential cell death as % cell viability was observed as 96.77 ± 1.59% (HT22 cell line) and 97.73 ± 1.87 (CTX TNA2 cell line). Even at the highest tested concentration at 50 µg/ml, there was a minor reduction in the cell viability as 87.83 ± 1.13% (HT22) and 88.75 ± 1.34% (CTX TNA2). Thus, overall cytotoxicity data revealed PSLN-2’s non-toxic, tissue-compatible nature to healthy brain/neuronal cells, which would promote the therapeutic applicability of PSLN-2 *in vivo*.

### *In vivo* neurobehavioral estimation

#### Y maze

**Fig. 5:**
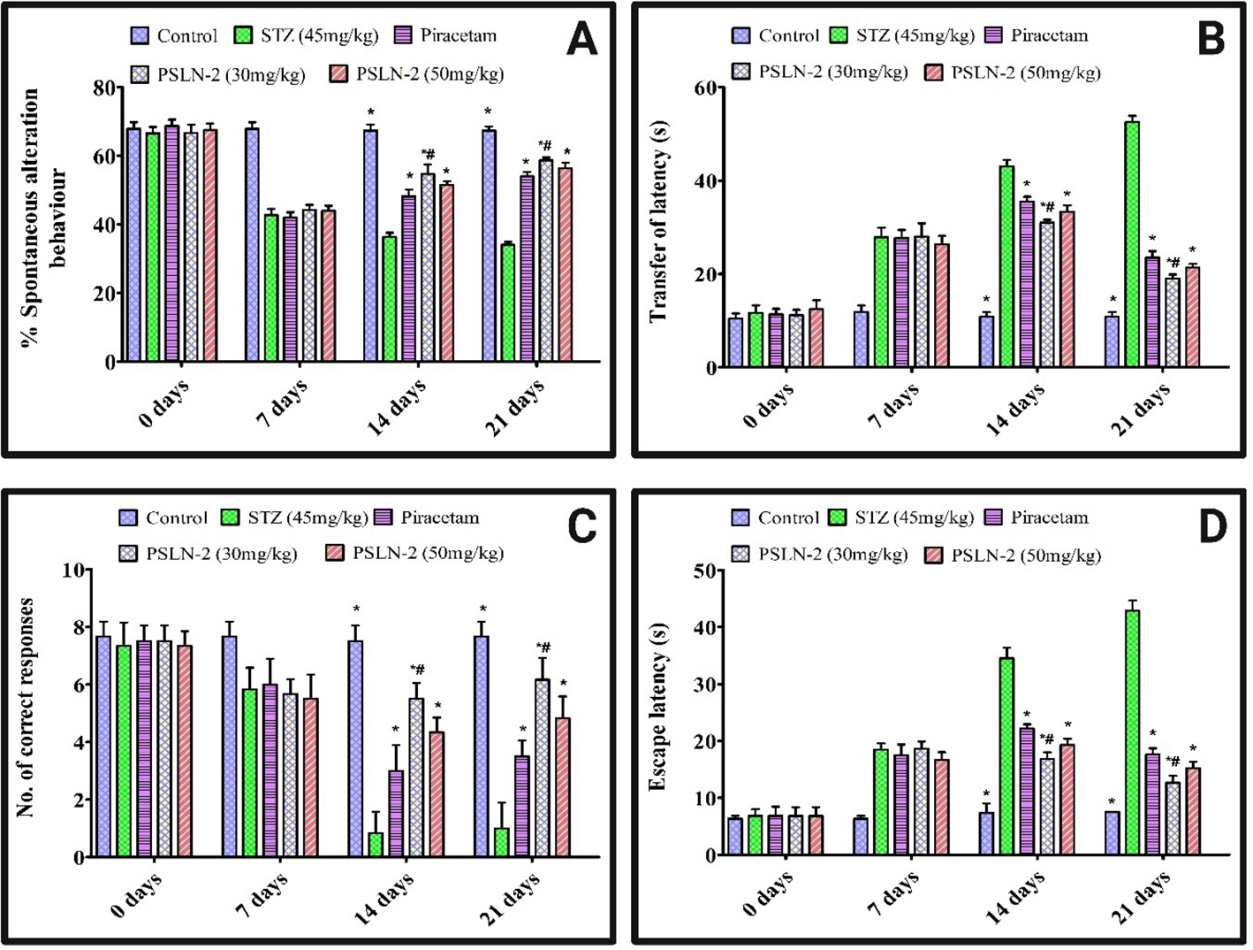
The effect of piracetam loaded solid lipid nanoparticle-2 (PSLN-2) 30 mg/kg and 50 mg/kg on percentage spontaneous alternation behaviour, transfer latency, number of correct responses, and escape latency in streptozotocin (STZ) induced cognitive impairment in rats using Y-maze (**A**), Elevated plus Maze (**B**), Radial Arm Maze (**C**), and Morris water maze (**D**). Values are expressed as mean ± SD where (n = 6), one-way ANOVA followed by Tukey’s post hoc *t*-test. *p < 0.05: Significant difference between the STZ group and all treatment groups (Control, Piracetam, PSLN-2 (30 mg/kg), and PSLN-2 (50 mg/kg)). ^#^*p* < 0.05: Significant difference between the Piracetam group and PSLN-2 (30 mg/kg) and PSLN-2 (50 mg/kg) groups

In the Fig. 5**A**, showed the % spontaneous alteration behaviour of rats at 7-day intervals for 21 days, (i.e. 0 day, 7 days, 14 days, and 21 days). % spontaneous alteration behaviour was significantly (*p* < 0.05) lowered by STZ on day 7 in all treatment groups as compared to control group. Piracetam, PSLN-2 (30 mg/kg and 50 mg/kg) on 14 and 21 day significantly (*p* < 0.05) increased the % spontaneous alteration behaviour as compared to STZ. Further PSLN-2 (30 mg/kg) showed a significant (*p* < 0.05) increase in the % spontaneous alteration behaviour as compared to the plain piracetam. Results overall showed the increase in % spontaneous alteration behaviour by PSLN-2 is not dose dependent.

#### Elevated plus maze

Rat’s transfer of latency at 7-day intervals across a 21-day period at 0, 7, 14, and 21 days is depicted in Fig. 5** B**. STZ significantly (*p* < 0.05) increased the transfer of latency on day 7 in all treatment groups as compared to control. Piracetam, PSLN-2 (30 mg/kg and 50 mg/kg) on 14 and 21 day significantly (*p* < 0.05) decreased the transfer of latency as compared to STZ rats. Further, PSLN-2 (30 mg/kg) showed a significant (*p* < 0.05) reduction in the transfer of latency as compared to the piracetam. However, the decrease in transfer of latency in case of PSLN-2 was found as dose independent.

#### Radial arm maze

A radial arm maze was utilized to examine the rats who had received STZ pre-treatment in order to determine the number of correct responses across a 21-day period at 0, 7, 14, and 21 days is depicted in Fig. 5**C**. STZ significantly (*p* < 0.05 decreased the number of correct responses on day 7 in all treated groups as compared to control group. Piracetam, PSLN-2 (30 mg/kg and 50 mg/kg) on 14 and 21 days significantly (*p* < 0.05) increased the correct responses as compared to STZ. Further, PSLN-2 (30 mg/kg) showed a significant (*p* < 0.05) increase in the correct responses as compared to the piracetam. However, the dose of PSLN-2 and corresponding response was not directly proportional.

#### Morris water maze

Rat’s escape latency at 7-day intervals across a 21-day period at 0, 7, 14, and 21 days is depicted in Fig. 5**D**. STZ significantly (*p* < 0.05) increased the escape latency on day 7 as compared to control group. However, in comparison to the STZ-induced group, the piracetam, PSLN-2 (30 mg/kg and 50 mg/kg) groups showed a substantial decrease in escape latency (*p* < 0.05) at day 14 and 21. Further, PSLN-2 (30 mg/kg) showed a significant (*p* < 0.05) alleviate in the escape latency as the rats spent more time in the target platform as compared to the piracetam treated rats.

### *In vivo* antioxidant and biochemical assessment

#### Effect of PSLN-2 on the SOD, CAT, and GSH levels in the rat brain

**Fig. 6:**
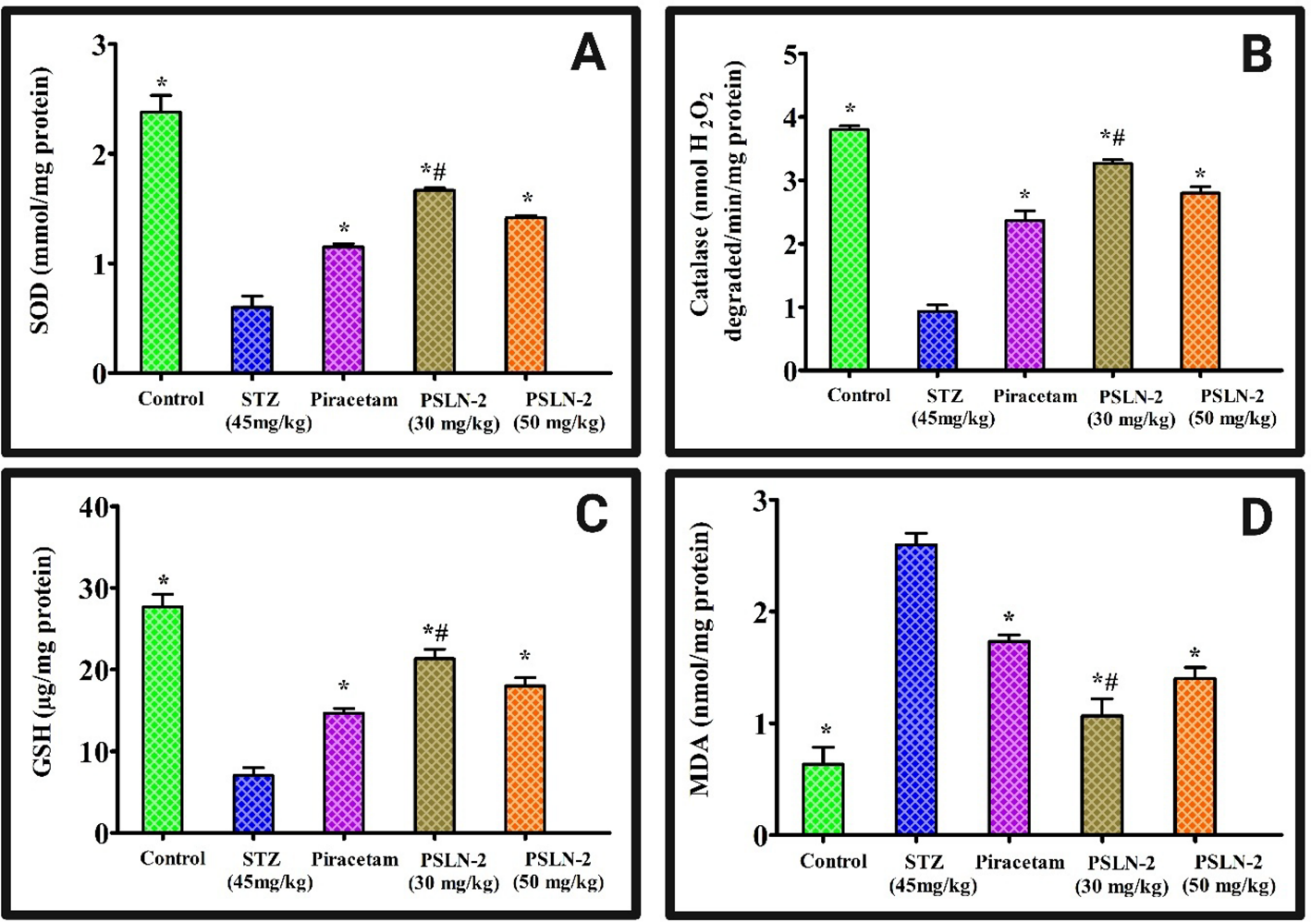
Antioxidant enzymatic activity **A** Superoxide dismutase (SOD) (mmole/mg protein), **B** Catalase (nmol H_2_O_2_ degraded/min/mg protein), and **C** Glutathione (GSH) (μg/mg protein) **D** Malondialdehyde (MDA) (nmole/mg protein) in brain of rats with streptozotocin (STZ) induced cognitive impairment after administration of piracetam and piracetam loaded solid lipid nanoparticles-2 (PSLN-2) (30 mg/kg and 50 mg/kg). Values are expressed as mean ± SD where (n = 3), one-way ANOVA followed by Tukey’s post hoc *t*-test. *p < 0.05: Significant difference between the STZ group and all treatment groups (Control, Piracetam, PSLN-2 (30 mg/kg), and PSLN-2 (50 mg/kg)). ^#^*p* < 0.05: Significant difference between the Piracetam group and PSLN-2 (30 mg/kg) and PSLN-2 (50 mg/kg) groups

The brain cells include two essential antioxidants, SOD and GSH, which reduce reactive oxygen species activity and oxidative stress markers [65]. Likewise, the cytosolic enzyme glutathione peroxidase facilitates the conversion of hydrogen peroxide into water, which is promoted by the ubiquitous antioxidant enzyme catalase. In comparison to control, STZ substantially (*p* < 0.05) reduced SOD, CAT, and GSH levels across all treatment groups in 21 days. In contrast to STZ, piracetam, PSLN-2 (30 mg/kg and 50 mg/kg) on 21 days markedly (*p* < 0.05) raised the activity of SOD, CAT, and GSH. Additionally, compared to piracetam, PSLN-2 (30 mg/kg) significantly (*p* < 0.05) increased SOD, CAT, and GSH activities (Fig. 6** A**–**C**).

#### Effect of PSLN-2 on the MDA levels in the rat brain

MDA is regarded as the indicator of oxidative stress, which is often brought on by the cell’s peroxidation of polyunsaturated fatty acids [66]. In 21 days, STZ substantially (*p* < 0.05) raised the MDA level in comparison to the control. In contrast to STZ, piracetam, PSLN-2 (30 mg/kg, and 50 mg/kg) on 21 days substantially (*p* < 0.05) decreased the MDA activity. Additionally, compared to piracetam, PSLN-2 (30 mg/kg) demonstrated a substantial (*p* < 0.05) decrease in MDA activity (Fig. 6** D**). However, PSLN-2 at 50 mg/kg did not show any significant reduction in MDA activity.

#### Nitrite level estimation

**Fig. 7:**
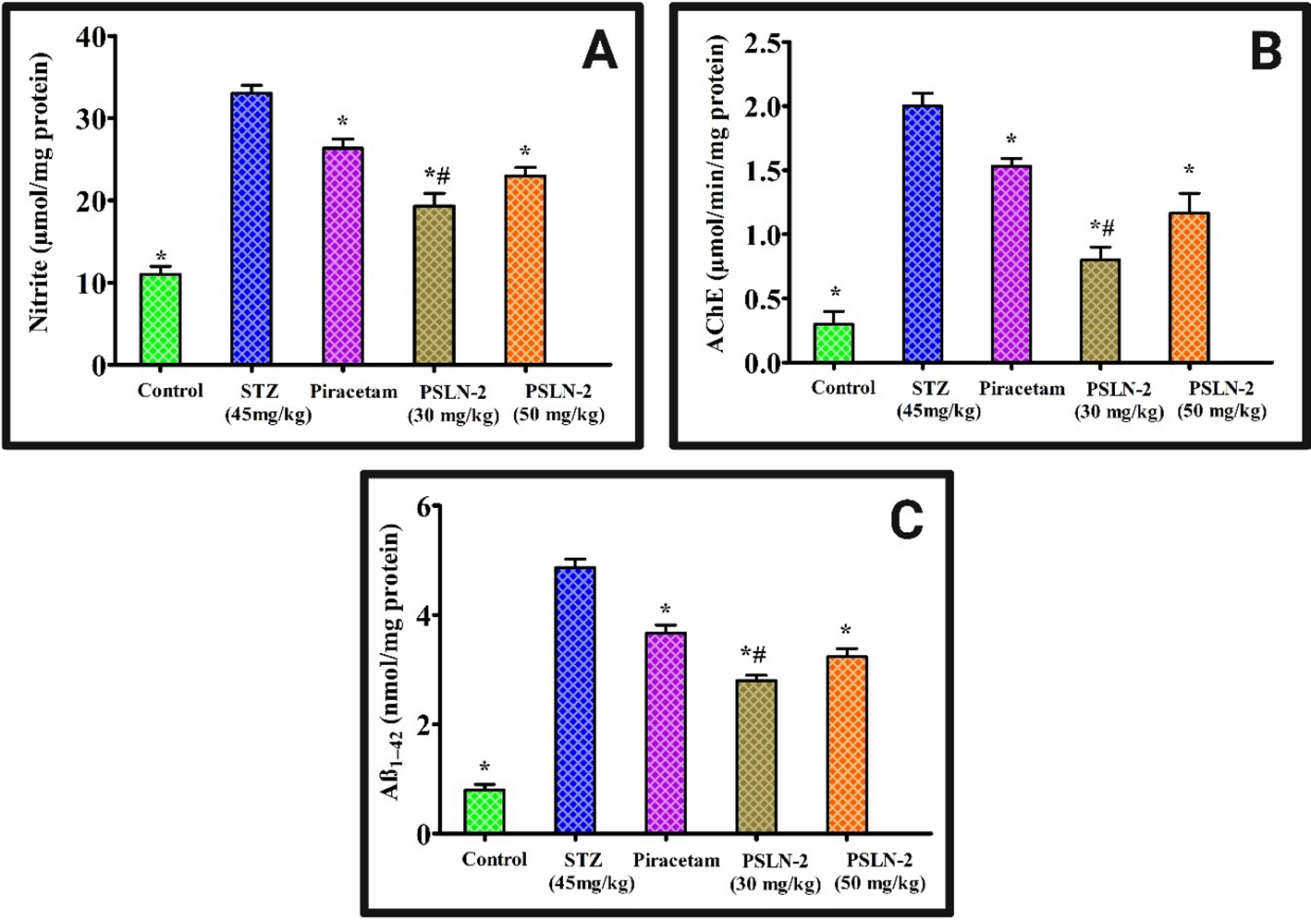
Estimation of **A** Nitrite (μmol/mg protein), **B** Acetylcholinesterase (AChE) level (μmol/min/mg protein), **C** Amyloid beta (Aβ_1-42_ ) (nmol/mg protein) content in streptozotocin (STZ) induced rats. Following administration of piracetam, piracetam loaded solid lipid nanoparticles-2 (PSLN-2) (30 mg/kg and 50 mg/kg) significant reduction in the protein contents was observed across all treatment groups. Values are expressed as mean ± SD where (n = 3), one-way ANOVA followed by Tukey's post hoc *t*-test. *p < 0.05: Significant difference between the STZ group and all treatment groups (Control, Piracetam, PSLN-2 (30 mg/kg), and PSLN-2 (50 mg/kg)). ^#^*p* < 0.05: Significant difference between the Piracetam group and PSLN-2 (30 mg/kg) and PSLN-2 (50 mg/kg) groups

The nitrite concentration in the brain is related to cognitive impairment [67]. The nitrite concentration in the brains of the STZ-induced rats was significantly (*p* < 0.05) higher than that of the control group after 21 days. However, as compared to STZ, the nitrite activity was considerably (*p* < 0.05) decreased by the administration of piracetam, PSLN-2 (30 mg/kg and 50 mg/kg) on days 21. Additionally, PSLN-2 (30 mg/kg) significantly (*p* < 0.05) decreased nitrite activity in comparison to plain piracetam (Fig. 7** A**).

#### AChE activity estimation

ACh is rapidly degraded in the brain as a result of increased AChE synthesis, which disrupts synaptic signalling and reduces cognitive function [68]. Thus, suppression of AChE improves cognitive function. At 21 days, the STZ-induced rat brains had considerably (*p* < 0.05) higher levels of AChE than the control group. The AChE content increase on days 21 was significantly (*p* < 0.05) reduced by the piracetam, PSLN-2 (30 mg/kg and 50 mg/kg) treatments. But PSLN-2 (30 mg/kg) significantly (*p* < 0.05) reduced AChE activity as compared to piracetam, PSLN-2 (50 mg/kg) treated groups **(**Fig. 7** B**).

#### *Aβ*_*1-42*_* content estimation*

Aβ content is strongly correlated with memory and cognitive disorders [69]. Aβ_1-42_ levels in the rat brain were considerably (*p* < 0.05) elevated by STZ treatment in the current investigation as compared to the control group at 21 days. The effects of STZ were considerably (*p* < 0.05) reversed following treatment with piracetam, PSLN-2 (30 mg/kg and 50 mg/kg) by downregulating the Aβ_1-42_ levels. When compared to piracetam, the PSLN-2 (30 mg/kg) demonstrated a substantial (*p* < 0.05) protective effect **(**Fig. 7** C**).

### Histopathological observation

**Fig. 8:**
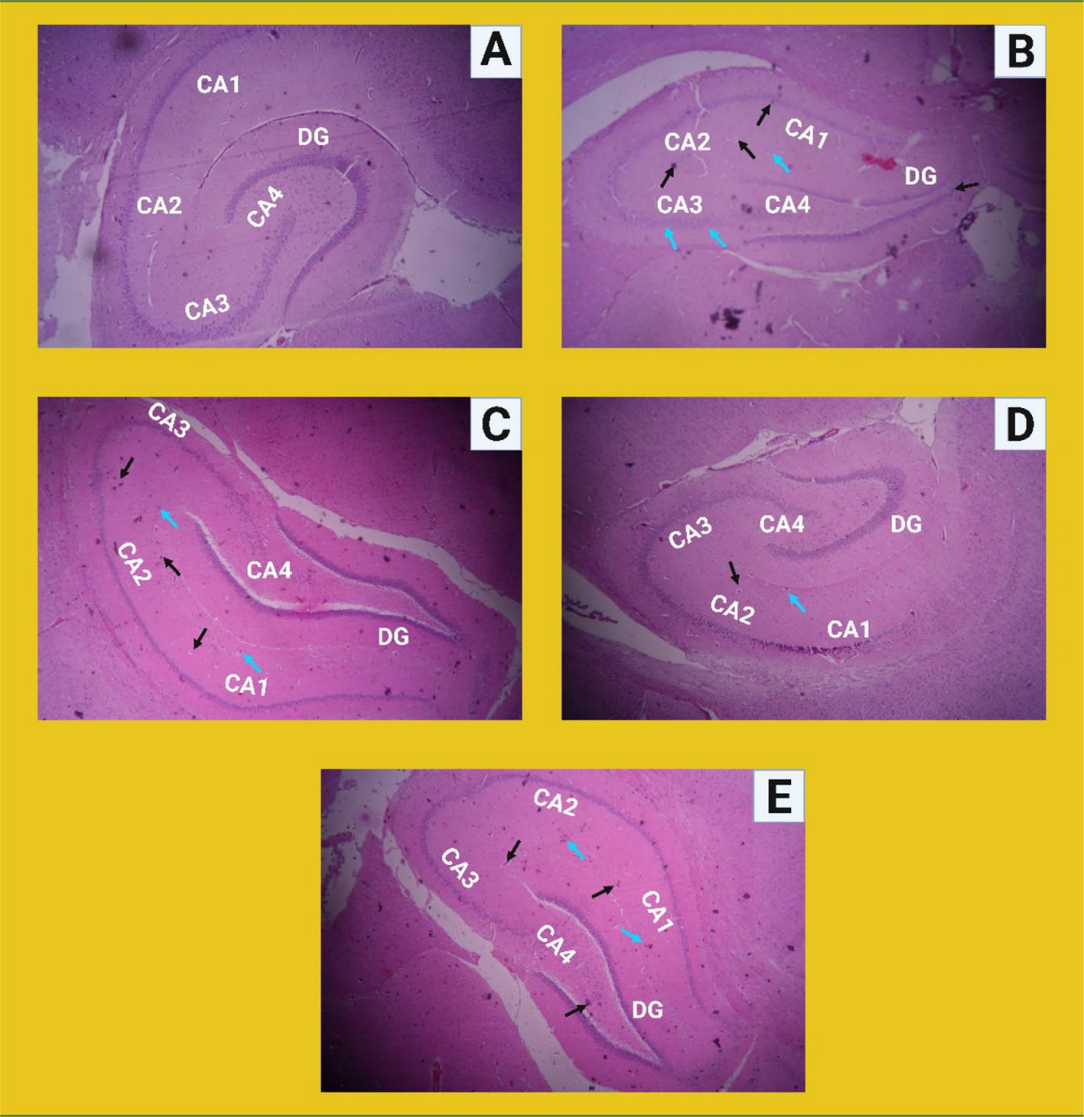
Histopathological representation showing the hippocampus [CA1, CA2, CA3, CA4 region and dentate gyrus (DG)] of the rat brain stained with dye haematoxylin and eosin (Scale = 400 μm, 10 × magnification). **A** The control group normal cell line structure of neuroglial cells with a thick nucleus free of neurodegeneration; **B** Streptozotocin (STZ) group showed that the disrupting hippocampal regions with neuroglial cell degeneration and significant necrosis (black coloured arrow) and pyknosis (sky coloured arrow); **C, E** Plain piracetam (100 mg/kg) and piracetam loaded solid lipid nanoparticles-2 (PSLN-2) (50 mg/kg) group showed that cells with less neurodegeneration, limited necrosis and pyknosis; **D** PSLN-2 (30 mg/kg) showed normal cytoarchitecture with organized neuronal cells and well-defined hippocampal area

Morphological changes in hippocampal cells indicates neurodegeneration. Neurodegeneration can lead to shrinking or swelling of hippocampal neurons, impacting their ability to transmit signals effectively. The hippocampal cells [CA1, CA2, CA3, CA4 area, and dentate gyrus (DG)] were significantly reduced in the brains of the STZ -treated rats (Fig. 8**)**. Healthy hippocampal sections with spherical nuclei, clear cytoplasm and normal neuronal cells free of neurodegeneration are shown in the control group. Large, dark-stained pyknotic neurons were seen in the STZ-induced groups. As the number of neurons decreased, the structure and appearance of the neurons deteriorated. PSLN-2 (30 mg/kg and 50 mg/kg) and piracetam treatment showed neuroprotective effects as observed from the recovery of damaged brain cells towards normal architecture. A distinct hippocampal region, a noticeable cytoarchitecture, and less pyknotic and necrotic cells without any neurodegeneration were all seen in PSLN-2 (30 mg/kg and 50 mg/kg) treated groups.

### Brain tissue pharmacokinetic analysis

**Fig. 9:**
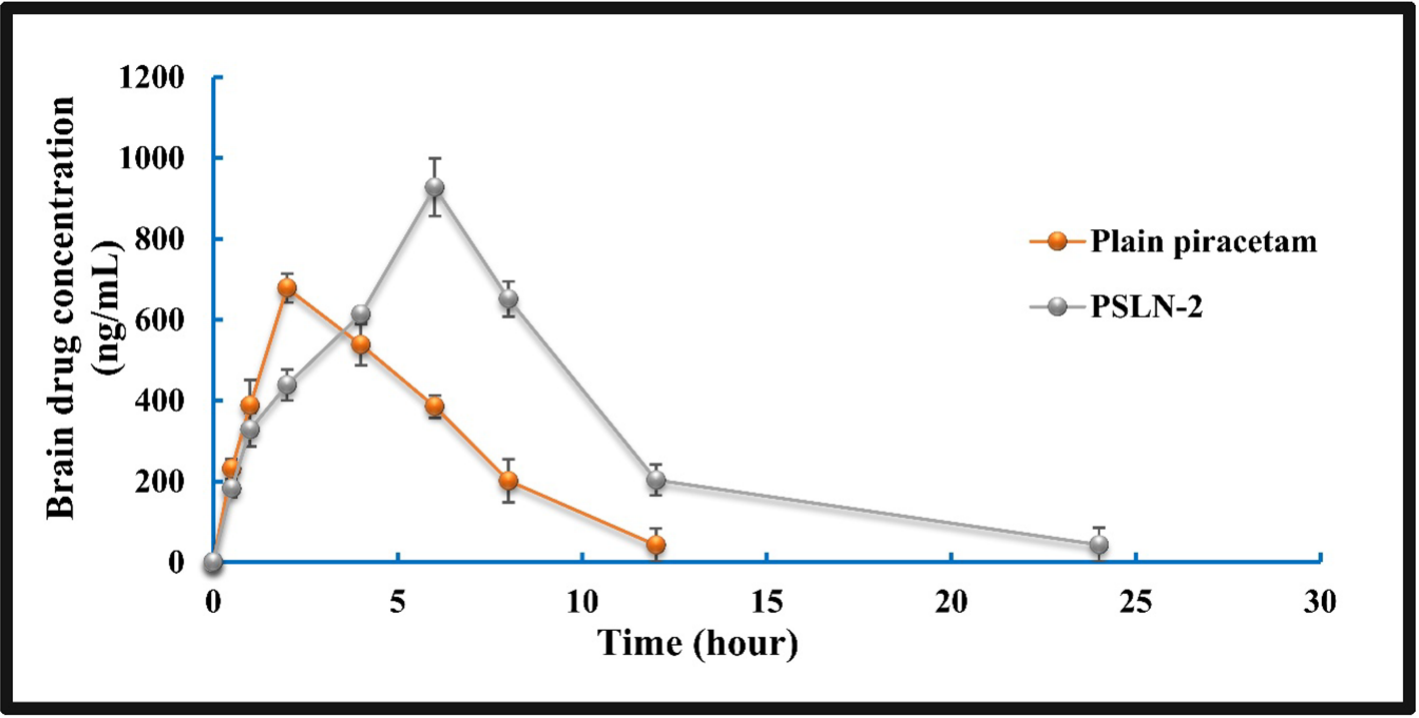
Brain pharmacokinetic analysis of piracetam loaded solid lipid nanoparticles-2 (PSLN-2)/plain piracetam post i.p. injection at different time points in experimental rats. Data show mean ± SD (n = 3)

Brain tissue PK study depicted a clear difference in the key parameters in terms of AUC, AUMC, MRT etc. among the plain piracetam, and PSLN-2 treated groups (Table 4). Following administration, the plain piracetam was detectable till 12 h, but at 24 h, the concentration of the drug was beyond the threshold limit of detection of LC–MS/MS (5 ng/mL). while the drug concentration from PSLN-2 was detectable till 24 h, signifying sustained release property of the formulation. AUC_0-t_ for plain piracetam was found as 5844.3 ± 189.2 ng ml^−1^ h^−1^, whereas the same was 18,351.7 ± 756.1for PSLN-2. Higher AUC for PSLN-2 signifies higher drug availability at brain tissue over the plain piracetam. Similarly, AUMC$_{{0 - \infty }}$ and MRT also followed the similar trend. The AUMC$_{{0 - \infty }}$ reported for PSLN-2 was 105,232.01 ± 911.7 ng h^2^ m^−1^, which was almost two folds higher than that of the plain piracetam (67,234.3 ± 811.5 ng h^2^ m^−1^). Higher MRT for the PSLN-2 suggests preferential improvement in piracetam accumulation in rat brain following nanoencapsulation. Further, a relatively higher clearance rate was found for plain piracetam (1.72 ± 0.8 L h^−1^) unlike PSLN-2 (0.71 ± 0.04 L h^−1^), improved permeation and retention of the experimental solid-lipid nanocarrier in brain, probably exploiting enhanced permeability and retention effect (Fig. 9).

**Table 4:** Estimation of brain pharmacokinetic parameter following i.p. bolus administration of plain piracetam/piracetam loaded solid lipid nanoparticles-2 (PSLN-2) in rats (n = 3)

Pharmacokinetic parameters	Plain piracetam	PSLN-2
C_max_ (ng/mL)	678.33	927.63*
AUC _0-t_ (ng h ml ^−1^)	5844.3 ± 189.2	18351.7 ± 756.1*
AUMC$_{{0 - \infty }}$ (ng h^2^ m^−1^)	67234.3 ± 811.5	105232.01 ± 911.7*
MRT (hour)	4.7 ± 0.2	9.2 ± 3.1*
V_d_ (L)	0.35 ± 0.4	1.76 ± 1.1*
Cl_t_ (L h^−1^)	1.72 ± 0.8	0.71 ± 0.04*

Data show mean ± SD (n = 3). AUC: area under the plasma concentration time curve; AUMC: area under the first moment curve; Cl_t_: clearance; MRT: mean residence time; V_d_: volume of distribution. *Data were significantly different (*p* < 0.05, students t’- test) where plain piracetam and PSLN-2 were compared

## Discussion

Cognitive impairment is increasingly acknowledged as an overlooked pathological condition of diabetes mellitus. The cognitive dysfunction linked to type-2 diabetes mellitus is accompanied by profound neurophysiological alterations and morpho-structural remodelling within the central nervous system. Previous studies indicate that approximately 20–70% of cognitive impairments are linked to type-2 diabetes mellitus [70, 71]. The study explored the neuroprotective efficacy of piracetam, a known nootropic agent, formulated into SLNs as a strategy to treat diabetic induced cognitive deficits. Recent evidence suggests that the lipid nanoparticles not only augment oxidative protection by stimulating the action of antioxidant enzymes but also have an inherent free-radical scavenging potential [72, 73]. Besides, their neuroprotective effect has also been supported in the mouse model of neurodegenerative diseases, thus making these nanoparticles outstanding carriers to counter neurodegenerative diseases [74].

Piracetam was found to be compatible with all the selected excipients and no physical/chemical drug-excipient interaction was there as depicted from FTIR, DSC, XRD studies. In FTIR, no significant shifting in characteristic spectral peaks of piracetam or SL/PLGA was detected in the physical blend or in the PSLNs. However, few minor shifting in the characteristic peaks of drug/excipient could be assigned to the weak physical interactions between the components such as Van der Waals, H-bond formation, electrostatic interaction etc., which might result in stable formulation structures.

Thermal analysis like DSC was used to evaluate the drug’s phase transition, melting point, and crystallization behaviour. Appearance of distinct endothermic peak in DSC thermogram of piracetam suggests sharp melting point and crystalline property. In contrast, the thermogram of PSLN-2, in view of diminished sharpness in endothermic peaks suggested amorphization of piracetam encapsulated in SLNs. Further, appearance of no new peaks or any shifting in the characteristic endothermic peaks of piracetam in PSLN-2 signifies stability and compatibility. XRD analysis reinforced the findings from the DSC study. The presence of sharp peaks confirmed the crystalline nature of piracetam, whereas the broader peaks with diminished peak height and width in PSLN-2 suggested amorphization. This indicates that piracetam existed in a molecularly dispersed or amorphous state within the SLN matrix. Furthermore, the XRD results confirm that piracetam retained its structural integrity after encapsulation. The transition to an amorphous form upon encapsulation would significantly improve the drug’s stability and bioavailability under physiological conditions.

Out of three selected formulations, PSLN-2 was selected as the suitable one based on % yield, %loading efficiency, size analysis data. Though, achievement of higher % drug encapsulation has been a challenging issue for nanoparticulate carriers, a reasonable loading of 20.1 ± 0.7% (w/w) for PSLN-2 can be considered significant. Higher yield (75.5 ± 1.5% w/w) for PSLN-2 further supports its feasibility towards industrial scale production. Further, it was observed that increase in the amount of piracetam in the formulations did not increase the loading percentage proportionately. Similar outcomes have been found in many such studies, where the amount of drug is not directly proportional to the %drug loading [75].

It has been already reported that the surface charge above + 30 mV to − 30 mV for the nanocarriers are crucial to form stable nanosuspension [34, 43]. A higher negative zeta potential (− 38.2 ± 0.3 mV) of PSLN-2 indicates its stability in dispersion form. Further, ultrasmall size of PSLN-2 would be suitable for BBB permeation as well as to avoid macrophage uptake *in vivo* [54]. A narrower and lower PDI in size analysis data indicates homogeneous particle distribution [76], which is again a desirable criteria of ideal nanoformulation intended for brain delivery.

SEM image of PSLN-2 depicted clear formation of nanostructures with spherical shape. Most of the lyophilized PSLN-2 was around 100 nm though few bigger size particles were discreetly observed within the sample. Overall the SEM data was in well agreement with average size data. Further absence of any aggregation or distortion in the nanoparticle structures signified proper formation which could be assigned to the standardized formulation ingredients and process parameters employed in the study. The surface morphology and topography of PSLN-2 was further substantiated by AFM, where a smooth and uniformly dispersed particle distribution with few amplitude projections indicated stable formulation characteristic.

A sustained piracetam release was observed from experimental PSLN-2 in (*In vitro*) over a 72 h release period. Result indicated that 68.11% of piracetam was released within the first 24 h, following which, the release rate plateaued over the subsequent 48 h, indicating an extended-release profile. This pattern is likely attributed to the long-term slow degradation of PLGA, where drug release is governed by a combination of diffusion and polymer erosion due to the slow hydrolysis of ester bonds [77]. Further, solid lipid carrier system and encapsulation of piracetam in the SLNPs matrix could be responsible for this release pattern. However, piracetam from marketed formulation showed a rapid drug release within a 6 h study period (98.7%). Clearly encapsulation of piracetam within solid lipid nanoparticle core imparted sustained release property. Sustained release of drug would reduce its dose, dosing frequency and dose-related side effects. This would in turn benefit AD patients for long term use. Further, the piracetam release data when fitted with various kinetic model showed best fitted with Higuchi model, which suggests that the encapsulated piracetam might be diffused through the porous matrix in one direction, following fickian diffusion process, i.e. rate of diffusion depends on the concentration gradient [78]. PSLN-2 was found stable under varying storage condition as depicted from the stability study. No significant changes in average size, PDI, zeta potential, or drug loading efficiency were observed even at 40 °C/75% RH. Overall the stability results indicated minor stress-related instability in PSLN-2 under recommended ICH storage conditions without significant compromise on formulation characteristics. Stable nature of PSLN-2 would be helpful for technology transfer and scalable method of production.

The DPPH and ABTS activity of PSLN-2 depicted strong anti-oxidant potential as compared to pure piracetam. The antioxidant activity of piracetam and PSLN-2 in scavenging DPPH free radicals was evident from the observed colour change of DPPH solution from purple to pale yellow. Lower IC_50_ value of piracetam and PSLN-2 depicted potential free radical scavenging activity. The scavenging effects of PSLN-2 were higher than those of plain piracetam in both studies. Overall the antioxidant activity followed the order as: ascorbic acid > PSLN-2 > piracetam. Presence of soy lecithin in PSLN-2 may synergistically contribute to the antioxidant effect of piracetam. The MTT test was used to establish the biocompatible and safety nature of the PSLN-2 on healthy brain/neuron cells. Overall cytotoxicity data revealed PSLN-2’s non-toxic, tissue-compatible nature to healthy neuronal cells, even at the highest tested concentration (50 μg/mL); as more than 85% of astrocyte and neuronal hippocampus cells were viable. Low cytotoxic nature of PSLN-2 would promote its application towards AD therapy.

Nitrosamines play a central role in triggering lipid peroxidation, DNA damage, mitochondrial impairment, nitrosative stress, activation of neuroinflammatory cytokines, and insulin resistance etc. [79, 80]. These effects collectively contribute to cellular deterioration and eventual cell death. STZ, a glucosamine nitrosourea compound, can induce cognitive decline by promoting neurodegeneration [81]. The Y-maze spontaneous alternation test serves as a reliable behavioural assay for evaluating spatial working memory, as well as the neurobiological modulators that influence its performance [82]. Similarly, in the radial arm maze, working memory function is operationally defined by the subject’s ability to enter each arm only once; re-entries are classified as working memory errors [83]. In the elevated plus-maze, transfer latency the latency period required for the animal to move from the open arm to an enclosed arm functions as a pharmacodynamic parameter indicative of acquisition and consolidation processes in memory [84]. The Morris water maze is a robust behavioural test for assessing long-term memory, widely employed to investigate pharmacological modulation of hippocampal-dependent cognitive processes [85]. Percentage of spontaneous alteration behaviour was reduced in STZ induced groups as depicted from Y-maze and the number of correct responses in the radial arm maze. In contrast, it significantly enhanced transfer and escape latency in the elevated plus maze and the Morris water maze, respectively. This validates the memory impairment caused by STZ [53]. Y maze, elevated plus maze, STZ’s impact was significantly (*p* > 0.05) reversed by piracetam, PSLN-2 (30 mg/kg and 50 mg/kg) with elevation in the spatial memory as well as acquisition and retention of memory. PSLN-2 (30 mg/kg) showed significant (*p* < 0.05) improvement in learning and memory than pure piracetam. The improved performance of PSLN-2 may be attributed to the higher availability of piracetam in rat brain following nanoencapsulation, which overcomes piracetam’s low penetration across the BBB in free form.

Oxidative stress, a key pathogenic factor in neurodegeneration and cognitive impairment, arises from excess generation of reactive oxygen and nitrogen species and inadequate antioxidant defence [86]. In type-2 diabetes mellitus, cognitive dysfunction is exacerbated by diminished levels of enzymatic antioxidants alongside mitochondrial dysfunction. This redox imbalance provokes hippocampal neuroinflammation, synaptic degeneration, and neuronal apoptotis or necrotic cell death. The alternation in the levels of various oxidative stress markers such as SOD, CAT, GSH, and MDA could be linked to the presence of free radicals and neuroinflammation [87, 88]. In the study, PSLN-2-treated groups showed a significant reduction in MDA levels and an elevation in SOD, CAT and GSH levels. Superior antioxidant activity of PSLN-2 than pure piracetam may be attributed to the nanoencapsulation and synergistic action of soy lecithin and piracetam.

PSLN-2’s neuroprotective impact in AD rat models is critically analysed by the evaluation of biochemical indicators of AD, such as nitrite content, AChE activity, and Aβ content. Nitrites are mutagenic substances that cause mitochondrial dysfunction, lipid peroxidation, insulin resistance, DNA damage, elevated inflammatory cytokines, cellular degeneration, and cell death [89–91]. AChE inhibition improves cognitive performance [92]. Furthermore, the main pathogenic feature of AD is the Aβ accumulation in the hippocampus area of the brain. In comparison to the control rats, the STZ-treated rats exhibited a substantial increase in nitrite content, AChE, and Aβ which is in agreement with earlier studies [56]. In contrast, plain piracetam/PSLN-2 (30 and 50 mg/kg) reversed these effects significantly. PSLN-2 (30 mg/kg) showed excellent neuroprotective action than piracetam and improved cognition.

Neuropathological analysis of brain tissues offers essential histological insights for evaluating neurodegenerative progression in AD models. In the present study, brain tissue sections from STZ induced rats exhibited characteristic neuropathological features such as neuronal atrophy, nuclear pyknosis, and marked hippocampal neurodegeneration. However, these neuropathological alterations were significantly reversed in PSLN-2 treated groups. The damaged brain tissues regained their normal histological architecture as evidenced from spherical nuclei, clearer cytoplasm and neuronal cells following PSLN-2 treatment.

Brain pharmacokinetic study was carried out to depict the availability of piracetam in brain following i.p. administration of plain piracetam/ PSLN-2. Data showed improved pharmacokinetic parameters in terms of AUC, AUMC, MRT and V_d_ for PSLN-2 than plain piracetam, which thus rationalized the *in vivo* efficacy results. Higher AUC straight forward denotes higher brain availability of the drug delivered through PSLN-2. Higher drug accumulation at brain in turn justified the improved therapeutic effectiveness observed in PSLN-2 treated group as compared to plain piracetam treated group. An increase in volume of distribution with decreased clearance rate for PSLN-2 suggests that the experimental lipoidal nanocarrier probably utilizing the enhanced permeability and improved retention effect might successfully bypass BBB and sustained for a preferential extended time period in the brain tissue, unlike the plain piracetam. Data overall indicated lipid nanocarrier mediated encapsulation brought significant modulation in the brain pharmacokinetic profile of piracetam.

Previous studies have shown that nanoformulations can produce enhanced pharmacological effects compared with the corresponding plain drug in Alzheimer’s disease models, often without a strict dose dependency [50]. Accordingly, piracetam (100 mg/kg) was selected as the standard reference owing to its well-documented cognitive-enhancing and neuroprotective properties. To assess the efficacy of the nanoformulation at reduced doses, PSLN-2 was evaluated at two lower dose levels (30 and 50 mg/kg) [93, 94]. At elevated nanoparticle concentrations, physicochemical interactions within biological matrices promote aggregation, primarily through protein corona and lipoprotein adsorption, thereby increasing hydrodynamic diameter, diminishing cellular internalization, and impairing tissue permeability. Such aggregation reduces therapeutic efficacy despite higher nominal dosing, as demonstrated with gold NPs, where clustered particles exhibited 25% lower uptake than monodisperse counterparts [95]. Ligand-functionalized NPs undergo receptor-mediated endocytosis governed by saturable, Michaelis–Menten-like kinetics; beyond receptor saturation, additional dosing fails to enhance uptake and may induce receptor recycling or downregulation [96, 97]. Furthermore, concentration-dependent modulation of the protein corona can sterically hinder targeting ligands, facilitating opsonin binding and redirection toward the mononuclear phagocyte system [98].

## Conclusions

The work depicted a facile, yet novel repurposing approach using piracetam as a model drug to ameliorate diabetic-induced AD pathology. Nanosized (within 100 nm), higher negative zeta potential, smooth surface texture, reasonable drug loading %, and sustained drug release (over 24 h) property of the experimental PSLN-2 rendered it suitable for *in vivo* application. Further, experimental PSLN-2 was stable within ICH recommended storage conditions, which would be beneficial for its futuristic large-scale optimization and during technology transfer. Preferential antioxidant activity (both *in vitro* and *in vivo*) with negligible cytotoxicity of PSLN-2 signifies its neuroprotective property without any detrimental effects on healthy brain/neuronal cells. The results were further echoed in histopathological analysis, where the AD rats showed a clear recovery of the cellular architecture and morphology following PSLN-2 treatment unlike the plain piracetam treated group or control groups. Following administration, PSLN-2 effectively ameliorated AD related behavioural dysfunctions and revived memory functions in diabetic-induced AD rats in a significant manner as compared to plain piracetam treated rats. In a similar vein, modulation in the expression of some of the crucial enzymes/proteins like CAT, SOD, GSH, AChE etc. were noticed in PSLN-2 treated group. Improved brain pharmacokinetic parameter for PSLN-2 further rationalized its therapeutic effectiveness in AD rats. Though the study underscored potentiality of piracetam as a potential anti-AD drug, but extensive research covering comparison of effectiveness analysis of PSLN-2 in other AD models along with *in vitro*-*in vivo* correlation studies are warranted. Overall outcome of the research may lay foundation stone towards furthering research on clinical applicability of PSLN-2.

## Supplementary Information

Supplementary Material 1.

## Data Availability

The authors so declare that there are no existing ethical concerns, all of the data in the work can be passed on for scientific purposes. No legitimate privacy, ethical, or safety concerns are violated by these data, nor do they compromise the confidentiality of human subjects. The data can be made available from corresponding author’s orcid id 0000-0002-8805-8682.

## Abbreviations

AD: Alzheimer’s disease
Aβ: Amyloid beta
AChE: Acetylcholineesterase
BBB: Blood-brain barrier
ACh: Acetylcholine
SLNs: Solid lipid nanoparticles
PSLNs: Piracetam loaded solid-lipid nanoparticles
STZ: Streptozotocin
PLGA: Polylactic-co-glycolic acid
PVA: Polyvinyl alcohol
SL: Soy lecithin
MTT: 3-(4,5-Dimethylthiazol-2-yl)-2,5-diphenyl-2H-tetrazolium bromide
DMEM: Dulbecco’s modified eagle medium
DPPH: 2,2-Diphenyl-1-Picrylhydrazyl
ABTS: 2, 2′-Azino-bis 3-ethylbenzthiazoline-6-sulfonic acid
EDTA: Ethylenediamine tetra acetic acid
DTNB: 5,5’-Dithiobis (2-nitrobenzoic acid)
TCA: Trichloroacetic acid
FTIR: Fourier transform infrared spectroscopy
DSC: Differential scanning calorimetry
XRD: X-ray diffraction analysis
SEM: Scanning electron microscopy
AFM: Atomic force microscopy
PBS: Phosphate buffered saline
SOD: Superoxide dismutase
GSH: Glutathione
MDA: Malondialdehyde
LC–MS/MS: Liquid chromatography–tandem mass spectrometry
AUC: Area under the curve
V_d_: Volume of distribution
AUMC: Area under the first moment curve
MRT: Mean residence time
Clt: Rate of tissue clearance
PDI: Poly dispersity index
