# Supplementary material for: Solid lipid nanoparticles enhance piracetam’s neuroprotective action in streptozotocin-induced cognitive dysfunction
Source: Discov Nano. 2026 Mar 29;21(1):89. doi: 10.1186/s11671-026-04528-3 (PMC13033468; doi:10.1186/s11671-026-04528-3)
Supplement: Supplementary file 1 — Supplementary Material 1. [file 11671_2026_4528_MOESM1_ESM.docx]

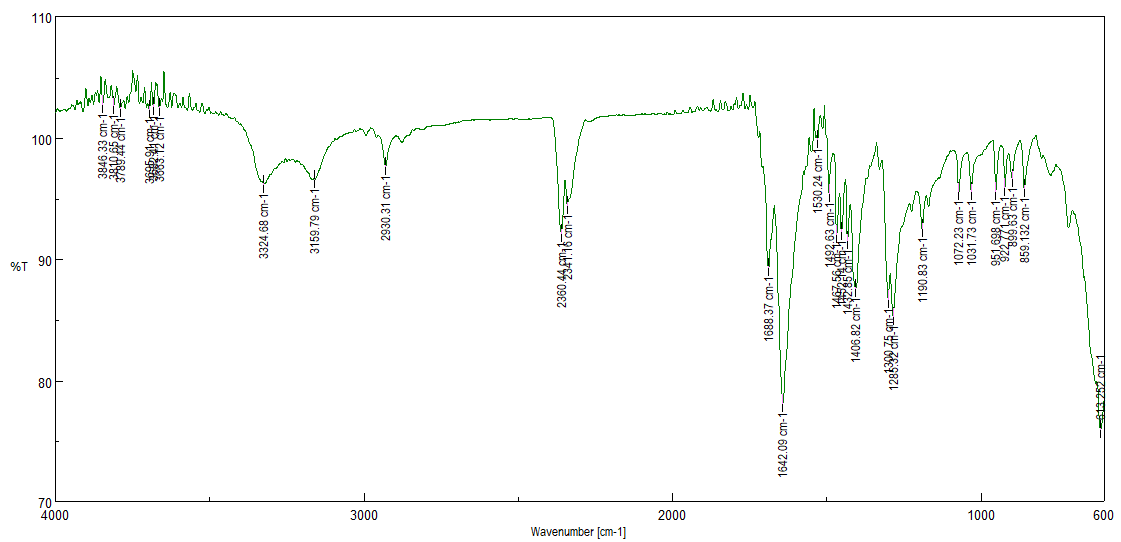


**Fig. S1** FTIR spectra of piracetam


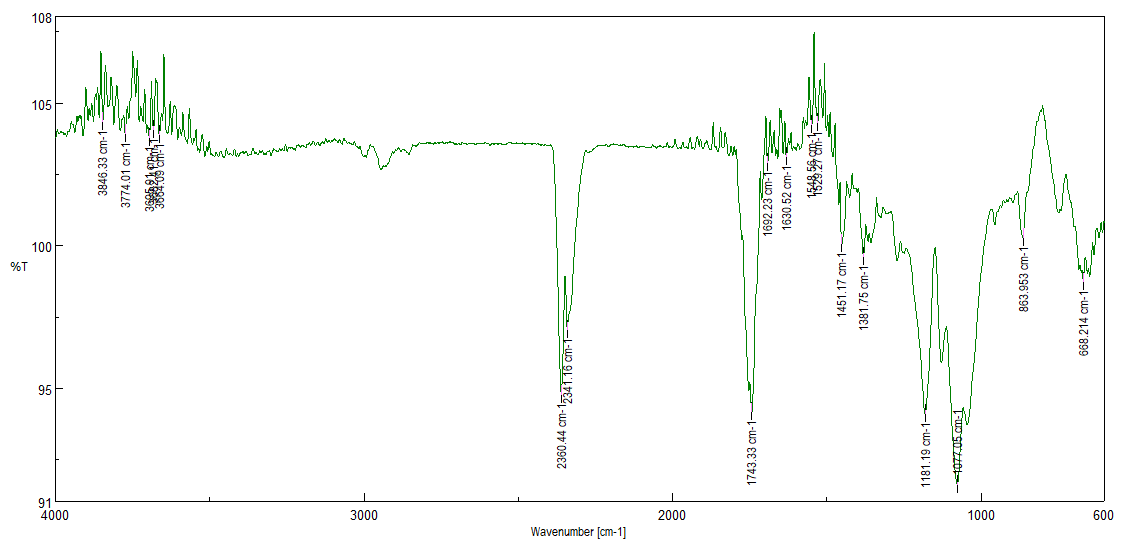


**Fig. S2** FTIR spectra of PLGA


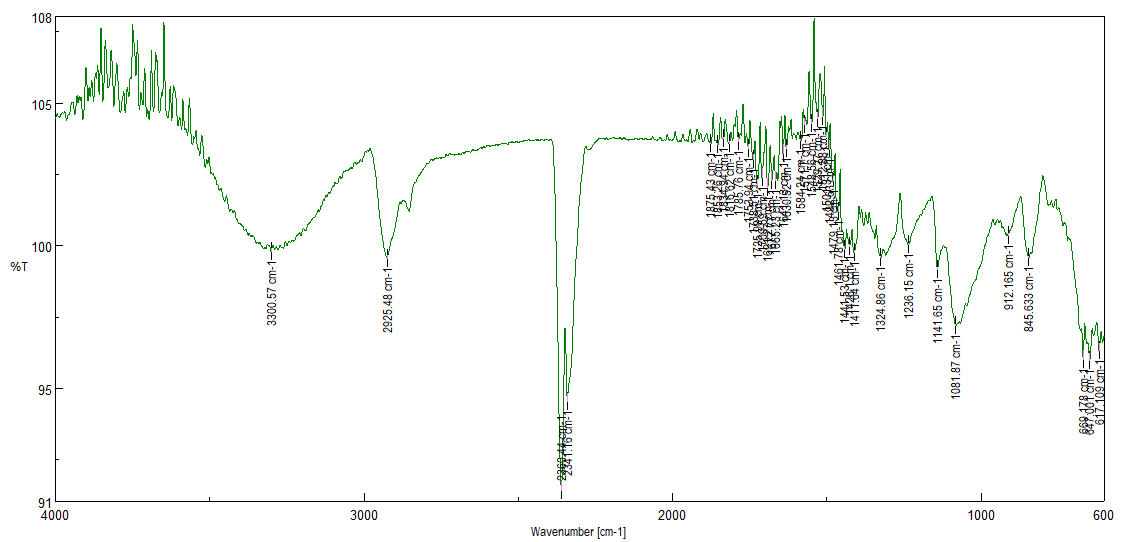


**Fig. S3** FTIR spectra of PVA


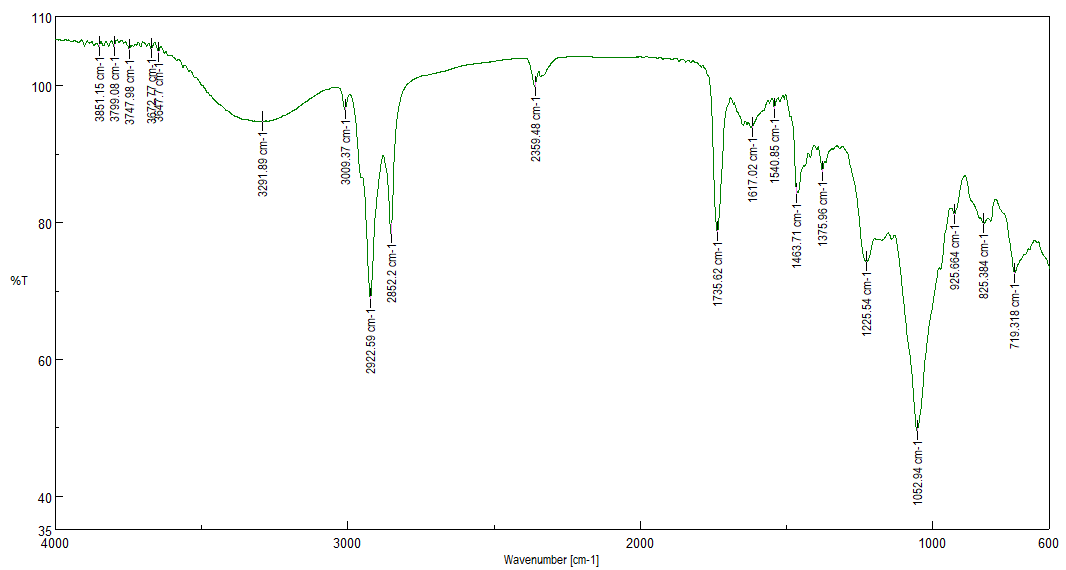


**Fig. S4** FTIR spectra of SL


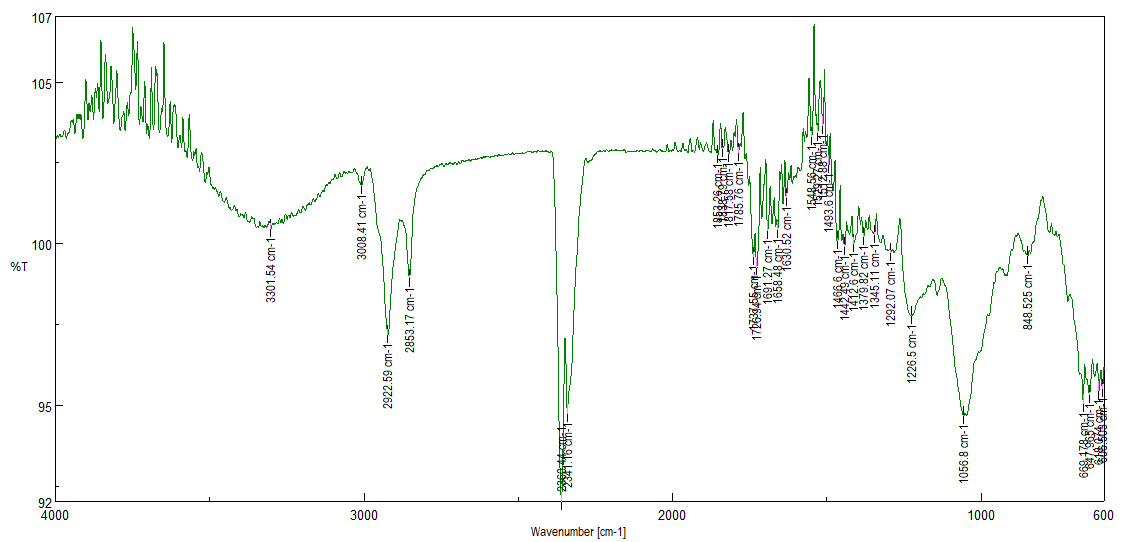


**Fig. S5** FTIR spectra of PM


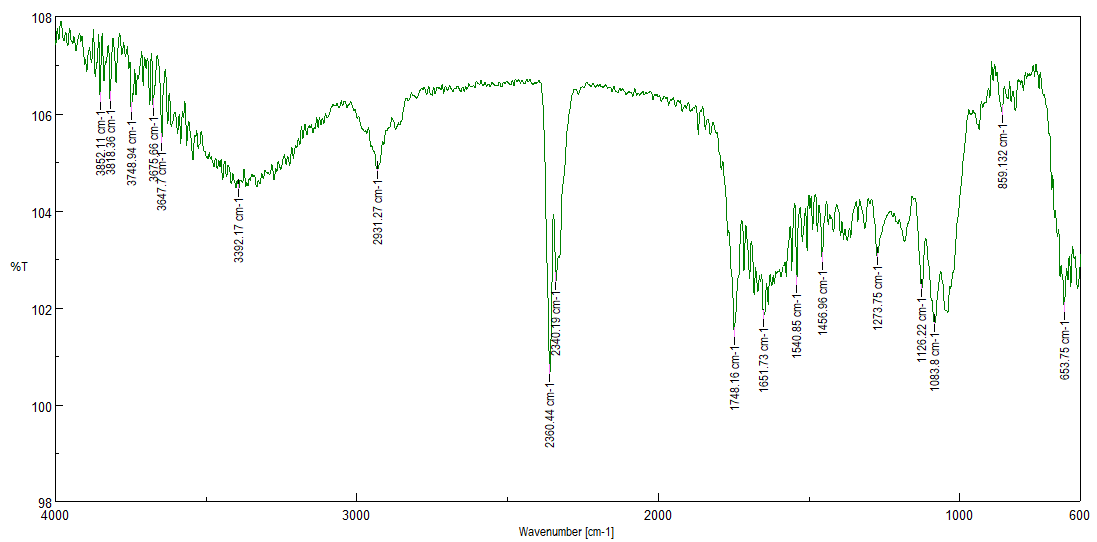


**Fig. S6** FTIR spectra of PSLN-2


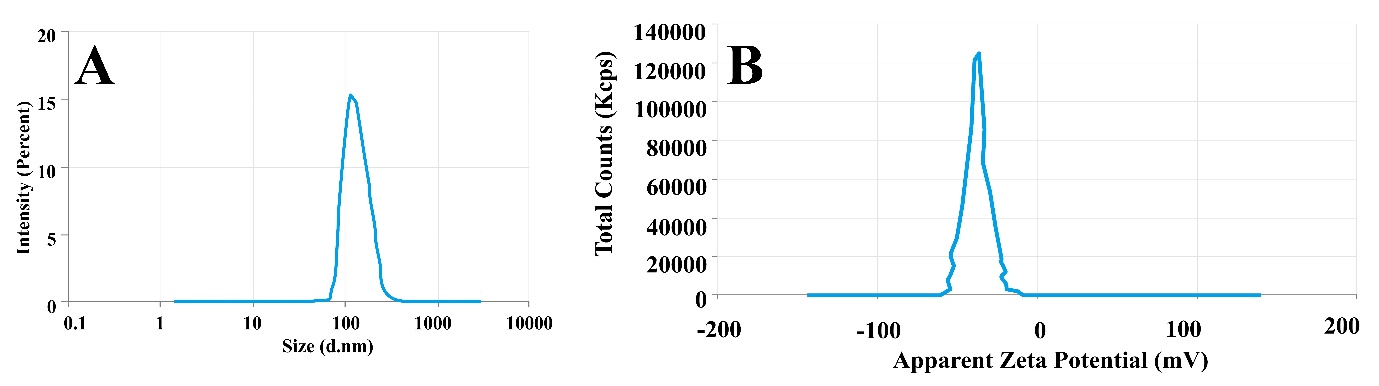


**Fig. S7** Average particle size, PDI and zeta potential of selected piracetam loaded solid lipid nanoparticles (PSLN-1)


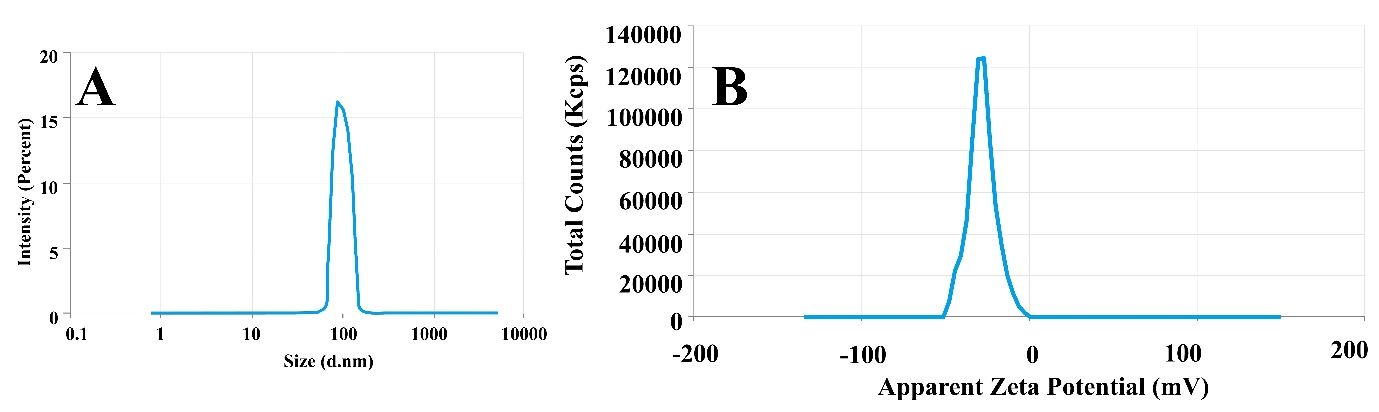


**Fig. S8** Average particle size, PDI and zeta potential of selected piracetam loaded solid lipid nanoparticles (PSLN-2)


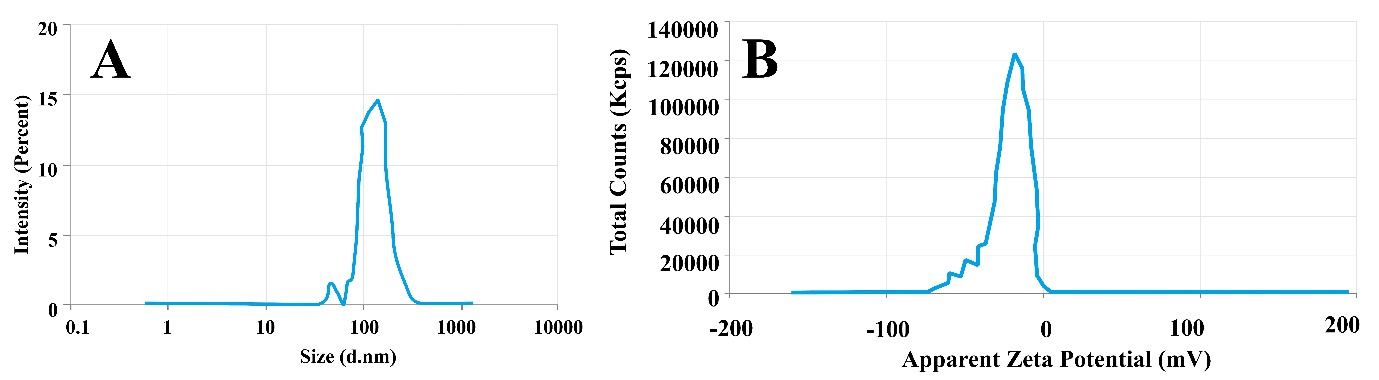


**Fig. S9** Average particle size, PDI and zeta potential of selected piracetam loaded solid lipid nanoparticles (PSLN-3)
